# Vestibular assessment in sudden sensorineural hearing loss: Role in the prediction of hearing outcome and in the early detection of vascular and hydropic pathomechanisms

**DOI:** 10.3389/fneur.2023.1127008

**Published:** 2023-02-15

**Authors:** Andrea Castellucci, Cecilia Botti, Silvia Delmonte, Margherita Bettini, Francesca Lusetti, Pasquale Brizzi, Rosanna Ruberto, Lisa Gamberini, Salvatore Martellucci, Pasquale Malara, Enrico Armato, Luigi Renna, Angelo Ghidini, Giovanni Bianchin

**Affiliations:** ^1^ENT Unit, Department of Surgery, Azienda USL – IRCCS di Reggio Emilia, Reggio Emilia, Italy; ^2^Ph.D. Program in Clinical and Experimental Medicine, University of Modena and Reggio Emilia, Modena, Italy; ^3^Audiology and Ear Surgery Unit, Azienda USL – IRCCS di Reggio Emilia, Reggio Emilia, Italy; ^4^ENT Unit, Santa Maria Goretti Hospital, Azienda USL di Latina, Latina, Italy; ^5^Audiology and Vestibology Service, Centromedico Bellinzona, Bellinzona, Switzerland; ^6^ENT Unit, SS. Giovanni e Paolo Hospital, Venice, Italy

**Keywords:** video head impulse (vHIT), vestibular evoked myogenic potentials (VEMPs), vestibulo-ocular reflex (VOR), labyrinthine ischemia, spontaneous nystagmus, sudden sensorineunal hearing loss, Menière's disease

## Abstract

**Introduction:**

Predicting hearing outcome in sudden sensorineural hearing loss (SSNHL) is challenging, as well as detecting the underlying pathomechanisms. SSNHL could be associated with vestibular damage since cochleo-vestibular structures share the same vascularization, along with being in close anatomical proximity. Whereas viral inflammations and autoimmune/vascular disorders most likely represent the involved aetiologies, early-stage Menière's disease (MD) can also present with SSNHL. Since an early treatment could beneficially influence hearing outcome, understanding the possible etiology plays a pivotal role in orienting the most appropriate treatment. We aimed to evaluate the extent of vestibular damage in patients presenting with SSNHL with or without vertigo, investigate the prognostic role of vestibular dysfunctions on hearing recovery and detect specific lesion patterns related to the underlying pathomechanisms.

**Methods:**

We prospectively evaluated 86 patients with SSNHL. Audio-vestibular investigation included pure-tone/speech/impedance audiometry, cervical/ocular-VEMPs, vHIT and video-Frenzel examination. White matter lesions (WML) were evaluated on brain-MRI. Patients were followed-up and divided into “SSNHL-no-vertigo,” “SSNHL+vertigo” and “MD” subgroups.

**Results:**

Hearing was more impaired in “SSNHL+vertigo” patients who exhibited either down-sloping or flat-type audiograms, and was less impaired in “MD” where low frequencies were mostly impaired (*p* < 0.001). Otolith receptors were more frequently involved than semicircular canals (SCs). Although the “SSNHL-no-vertigo” subgroup exhibited the lowest vestibular impairment (*p* < 0.001), 52% of patients developed otolith dysfunctions and 72% developed nystagmus. Only “MD” subjects showed anterior SC impairment and upbeating spontaneous/positional nystagmus. They more frequently exhibited cervical-VEMPs frequency tuning (*p* = 0.036) and ipsilesional spontaneous nystagmus (*p* < 0.001). “SSNHL+vertigo” subjects presented with more frequently impaired cervical-VEMPs and posterior SC and with higher number of impaired receptors (*p* < 0.001). They mainly exhibited contralesional spontaneous and vibration-induced nystagmus (*p* < 0.05) and only they showed the highest WML score and “vascular” lesion patterns (*p* < 0.001). Concerning the outcomes, hearing was better in “MD” and worse in “SSNHL+vertigo” (*p* < 0.001). Hearing recovery was mostly affected by cervical-VEMPs impairment and the number of involved receptors (*p* < 0.05). Patients with “vascular” lesion patterns presented with the highest HL degree and WML score (*p* ≤ 0.001), while none of them exhibited a complete hearing recovery (*p* = 0.026).

**Conclusions:**

Our data suggest that vestibular evaluation in SSNHL can provide useful information on hearing recovery and underlying aetiologies.

## 1. Introduction

Sudden sensorineural hearing loss (SSNHL) represents a sensorineural hearing impairment ≥30 dB over at least three contiguous frequencies on audiometry occurring within a 72-h time span. Although it mostly occurs unilaterally with a higher incidence in the 3rd–5th decades of life, SSNHL can affect patients of any age, whereas it might rarely present in both ears (simultaneous or sequential) (1). According to the literature, SSNHL is often accompanied by additional inner ear symptoms (vertigo, dizziness, tinnitus and aural fullness) while the estimated prevalence of the sole vestibular symptoms is 30%−60%. SSNHL is thought to represent an inner ear disorder in the majority of cases, despite infarction in the territory supplied by the vertebra-basilar system might result in sudden deafness with or without vertigo (2, 3). Despite an accurate history taking, including the evaluation of specific risk factors, and the use of various diagnostic tests, including laboratory tests and brain magnetic resonance imaging (MRI), a definite aetiologic factor is not identified in the vast majority of patients. This is the reason why SSNHL is mostly considered an idiopathic condition. While systemic diseases, neoplasms, neurological and otologic disorders, toxic, and traumatic agents represent some of the identifiable causes, some etiopathogenetic theories have been proposed for idiopathic SSNHL, including viral inflammations, immune-mediated mechanisms and vascular damage to the inner ear (4–6). Epidemiologic studies highlighting a close relationship between SSNHL and vascular disorders and, on the other hand, the evidence that patients with SSNHL exhibit a higher risk to develop cardiocerebrovascular diseases support the latter theory (7, 8). It has also been documented a higher arterial stiffness and a higher prevalence of leukoaraiosis on brain MRI in subjects with SSNHL compared to controls, further strengthening a vascular hypothesis (9, 10). In fact, leukoaraiosis represents a diffuse alteration of the periventricular and subcortical white matter, correlated to the small vessel disease and characterized by hyperintensity of the white matter lesions (WML) on T2-weighted images (11). Nevertheless, also Menière's disease (MD), which represents a multifactorial and heterogeneous disease likely due to an underlining endolymphatic hydrops (EH) as documented by histopathological, radiological and physiological studies (12–17), can present with isolated SSNHL affecting low frequencies as first clinical manifestation of. In fact, only a minority of MD patients debuts with the full clinical triad, including concomitant ictal vertigo and tinnitus, since hearing could exhibit delayed fluctuations and vestibular symptoms might occur only at a later stage (18, 19). Since auditory function in MD behaves differently from other SSNHL, as it tends to recover spontaneously and better than other conditions, some Authors usually prefer to rule out patients with low-frequency HL from inclusion criteria of idiopathic SSNHL to avoid bias. Nevertheless, it is well-known how the first episode of acute HL in MD might not only affect the low tones, but also middle and/or higher frequencies (20, 21). In addition, it has been described that only part of subjects with low-tone SSNHL develops a clinical picture consistent with MD over time and, on the other hand, that an additional subgroup of patients with idiopathic SSNHL later develop MD in the ipsilateral ear (ipsilateral delayed EH) (19, 22–24). Therefore, considering low-frequency SSNHL as a certain manifestation of MD “*a priori*” does not seem to represent a safe approach.

Steroid therapy represents the current mainstay of treatment for SSNHL. Additional treatment strategies include antiherpetic therapy, rheologic agents, diuretics, hyperbaric oxygen therapy, fibrinogen/LDL-apheresis and intratympanic injections. Nevertheless, spontaneous hearing recovery has also been reported in up to 65% of patients with SSNHL (1, 5, 6, 25).

Predicting hearing outcome in SSNHL is still challenging. Patient's age, configuration and degree of HL, comorbidities and time between onset of symptoms and treatment have been widely accepted as the most influencing factors on hearing outcome (5, 6, 26). Additionally, the presence of vestibular symptoms and the involvement of the vestibular end-organs have been considered supplementary poor prognostic factors, as they are usually associated with a greater inner ear damage, deeper HL and worse hearing recovery (27–37). Several studies investigating the clinical significance of vertigo and data obtained from vestibular tests have been conducted, yielding some conflicting results (26–51). One of the reasons for these discrepancies is that vertigo is not a specific disease entity but a symptom, and similar vestibular abnormalities can result from different pathologies not sharing the same etiologies, leading to heterogeneous group of patients. Theoretically, given the various embryological and anatomical factors making the inner ear (cochlear and vestibular partitions) an anatomo-physiological unity, it is possible to expect different lesion patterns depending on the underlying etiologies in case of SSNHL. Thanks to the combined use of recently introduced tests for vestibular assessment, such as the video-Head Impulse Test (vHIT) (52) and vestibular-evoked myogenic potentials (VEMPs) (53), assessing semicircular canal (SC) and otolith reflexes, respectively, and both branches of the vestibular nerve (VN), it has become possible to assess overall labyrinthine receptors and afferents even in acute setting, outlining lesion patterns peculiar to specific pathomechanisms (32, 54–62). In fact, while some patterns closely overlapped sensors innervated by the two divisions of the vestibular nerve likely due to viral neuritis (54, 55, 63, 64), inner ear ischemia has been considered the most likely mechanism for those patterns overlapping the territories supplied by the terminal branches of the internal acoustic artery (IAA) (57–59, 61–63, 65–68).

It is well-known how an early treatment could beneficially influence hearing outcome and symptoms recovery in SSNHL, therefore the detection of the possible etiology could play a pivotal role in orienting the most appropriate treatment. In addition, since it has been demonstrated how labyrinthine vascular lesions may precede a major stroke involving posterior fossa structures, clinicians' awareness toward the eventuality of an inner ear ischemia should be risen (69, 70). The aim of this study is to address the following questions, comparing our results with the pertinent literature:

- Identifying the extent of vestibular dysfunction quantitatively in patients affected by SSNHL with or without vertigo;
- Addressing the prognostic factors, including instrumental data and oculomotor findings, for SSNHL over 6 months;
- Identifying peculiar lesion patterns associated to specific pathomechanisms and etiologies underlying SSNHL.

## 2. Materials and methods

### 2.1. Ethical statement

This study was approved by our Institutional Review Board (Area Vasta Emilia Nord, approval number 238/2020/OSS/AUSLRE) and was conducted according to the tenets of the Declaration of Helsinki (2002). Written informed consent was obtained from each patient enrolled in the study.

### 2.2. Cohort and study design

We prospectively enrolled all consecutive adult patients (≥18 years old) with unilateral SSNHL who were admitted in Day Service of the Audiology and Ear Surgery Department of our Institution from January 2020 to June 2021. The diagnosis of SSNHL was based on unilateral SSNHL of more than 30 dB at a minimum of 3 consecutive frequencies over a period ≤ 72 h (1) occurred within the previous 30 days. All patients with onset of symptoms ≤ 30 days were included in the study. All patients who were admitted after more than 7 days from symptoms onset had already started an uneventful treatment with oral steroids. Subjects with previous HL in the same ear were excluded to avoid possible debate about the assessment of outcomes. All patients were treated according to the current therapeutic protocol for SSNHL in our Institution and received the same detailed work-up within 1 week from admission, including history taking, blood laboratory tests, pure tone audiometry, impedance audiometry, speech audiometry, video-Frenzel examination, VOR-gain assessment for all semicircular canals with the vHIT and both cervical and ocular-VEMPs for air-conducted (AC) sounds. Brain gadolinium-enhanced magnetic resonance imaging (MRI) was scheduled over a 3-month period in all cases, whereas temporal bones high-resolution computed tomography (CT) scan was performed only if needed in selected cases. Clinical records were collected and analyzed.

### 2.3. History taking and blood laboratory tests

Accompanying vestibular symptoms (acute vertigo and/or unsteadiness) and cochlear symptoms (aural fullness and/or tinnitus) were investigated, along with neurologic, endocrinologic and rheumatologic comorbidities. Each patient was investigated for precise cardiovascular risk factors [arterial hypertension, hypercholesterolemia, previous acute cerebrovascular or heart diseases, diabetes mellitus, body mass index (BMI) >25, smoke habits] and the sum of these factors (“0” absence, “1” presence) was calculated to assess the “cardiovascular risk score” for statistical purposes. Blood laboratory test included white blood cells count, PCR, TSH and biochemical assays for well-known biomarkers for cardiovascular disease associated to SSNHL (fibrinogen, homocysteine, total and LDL cholesterol, triglycerides, Apolipoprotein A1, Apolipoprotein B, Apolipoprotein A1/B ratio, Lipoprotein) (71). In case of at least one out-of-range laboratory assay among biomarkers for cardiovascular disease, the patient was assigned “+1” in the “cardiovascular risk score.” Virological screening test including IgM and IgG antibodies against Herpes Simplex virus (HSV) type 1 and type 2, Herpes Zoster virus (HZV), and Cytomegalovirus (CMV) was also conducted.

### 2.4. Audiometry

Pure-tone audiometry was performed over the frequency range of 125–8,000 Hz for air-conduction (AC) and 250–4,000 Hz for bone-conduction (BC) in a soundproof room using standard clinical procedures, once normal status of tympanic membranes and external auditory meatus was ascertained on micro-otoscopic examination. Appropriate masking was used for BC testing and, when needed, for AC. The pure tone average (PTA) was calculated as the average of the BC thresholds of the four most impaired contiguous frequencies. Morphologies of audiometries were categorized as low-frequency, high-frequency or flat-type depending on the most affected tones. Audiometries were also classified according to the HL severity in four categories: “mild” (PTA ≤ 40 dB), “moderate” (PTA >40 and ≤ 70 dB), “severe” (PTA >70 and ≤ 90 dB) and “profound/anacusis” (PTA > 90 dB). In case of anacusis, PTA of 120 dB was assigned for statistical purposes. Standard tympanometry with a 226 Hz probe tone and ipsi/contralateral acoustic reflexes were administered to all patients. Speech audiometry with lists of disyllabic words was imparted on both ears to assess the words recognition score (WRS).

### 2.5. Video-Frenzel examination

Eye movements were recorded with a monocular ICS video-oculographic system (GN Otometrics, Denmark) on admission. Preliminary bedside oculomotor testing including smooth pursuit, saccades, vergence and skew deviation was assessed to rule out central nervous system (CNS) abnormalities. Horizontal, vertical, and torsional components of nystagmus were qualitatively assessed. Horizontal (right/leftbeating), vertical (up/downbeating) directions of nystagmus, and torsional (right/left) components were described from the patient's point of view. Bedside-examination included the assessment of spontaneous (SN), gaze-evoked and positional nystagmus (PN) evoked by the supine head-roll test, Dix-Hallpike positionings on both sides and straight head-hanging position. SN was classified according to the predominant features: absent, beating contralesionally (i.e., paretic nystagmus) or ipsilesionally to the side with SSNHL (i.e., either irritative or recovery nystagmus, depending on cases), upbeating and downbeating. Conversely, PN was classified in absent, either geotropic/apogeotropic (in case it could only be detected by positioning the patient's head on one side) or bigeotropic/biapogeotropic (in case it was direction-changing, i.e., detected by positioning the patient's head on both sides) at the supine head roll-test, either persistent upbeating/downbeating or paroxysmal upbeating/torsional consistent with ipsilesional benign paroxysmal positional vertigo (BPPV) at the Dix Hallpike and straight head hanging positions. Skull vibration, head-shaking and hyperventilation tests were conducted with the patient upright. Vibration-induced nystagmus (VIN) was elicited applying a hand-held 100-Hz vibrator (VVIB 100 Hz Synapsys, France) to both mastoids for at least 15 s. VIN was considered reliable only if vibrations in both mastoids resulted in the same oculomotor pattern. Then, 30 cycles of passive head rotations were imparted at a rate of 1–2 Hz with head tilted 30° forward in the plane of the HSC and the head-shaking nystagmus (HSN) was evaluated in the 30 s following the test. Finally, the patient was instructed to hyperventilate deeply for 40 s, taking about one breath per second, and hyperventilation nystagmus (HVN) was evaluated in the following 30 s. In patients without SN, we considered as pathologic response the onset of sustained unidirectional nystagmus (horizontal or vertical/torsional). In cases with SN, either a sustained increase, an inhibition or an inversion of nystagmus, or even a modification of SN plane were accepted as pathologic responses. SN, VIN, HSN, and HVN were classified for statistical purposes in absent, ipsilesional and contralesional to the ear with SSNHL, upbeating and downbeating.

### 2.6. vHIT

The vHIT was performed on admission to evaluate the high-frequency vestibulo-ocular reflex (VOR) gain for each semicircular canal (SC), using an ICS video-oculographic system (GN Otometrics, Denmark). Passive, unpredictable 150°-250°/s and 3,000°-5,000°/s^2^ head impulses were delivered manually on the plane of the horizontal and vertical SCs while the patient was asked to keep looking at an earth-fixed target, according to the standard protocol (52). At least 15 stimuli were delivered for stimulating each SC and averaged to get the corresponding mean VOR-gain. VOR-gain values <0.8 for HSC and <0.7 for ASC and PSC with corrective saccades (overt and/or covert) were considered pathological. Data corresponding to VOR-gain for the horizontal (HSC), anterior (ASC) and posterior SC (PSC) for each pathologic ear were considered in statistical analyses.

### 2.7. VEMPs testing

Cervical and ocular VEMPs (cVEMPs and oVEMPs, respectively) for AC sounds were recorded using 2-channel evoked potential acquisition systems (Viking, Nicolet EDX, CareFusion, Germany) with surface electrodes placed according to standardized criteria (53). Potentials were recorded delivering tone bursts (starting intensity: 100 dB nHL, frequency: 500 Hz, duration: 8 ms, stimulation rate: 5 Hz). Recording system used an EMG-based biofeedback monitoring method to minimize variations in muscles contractions and VEMPs amplitudes. A re-test was performed for each stimulus to assess reproducibility. The first biphasic responses on the ipsilateral sternocleidomastoid muscle (p13-n23) for cVEMPs (ipsilateral response) and under the patient's contralateral eye (n10-p15) for oVEMPs (crossed response) were analyzed by calculating the peak-to-peak amplitude. Inter-aural amplitude difference between the ipsilesional (Aipsi) and contralesional ear (Acontra) to SSNHL were calculated with the asymmetry-ratio (AR): (Acontra – Aipsi) / (Acontra + Aipsi) × 100. Otolith sensors on the pathologic side were considered damaged if potentials resulted in AR ≥33% for both cVEMPs and oVEMPs, according to our normative data and to literature references (53), or in cases of bilaterally absent responses. Threshold was then obtained decreasing in steps of 10 dB from 100 dB nHL; the lowest stimulus intensity resulting in a clear and repeatable biphasic wave was considered as threshold. cVEMPs were then tested with 100 dB tone bursts at 1 kHz bilaterally: frequency tuning was considered “positive” if the difference between the amplitude of potentials obtained at 1 kHz and the amplitude at 500 Hz was ≥ 0 μV (14).

### 2.8. Imaging

Brain gadolinium-enhanced MRI (1.5 Tesla) was completed by standard protocols for posterior fossa visualization. The extent of leukoaraiosis was assessed with the Fazekas scale (11) on T2-weighted images or FLAIR sequences and classified in four stages according to presence, size, and confluence of periventricular and deep WML: 0 “absent,” 1 “foci,” 2 “beginning confluent” and 3 “large confluent areas.”

### 2.9. Treatment

According to the current uniform treatment protocol for SSNHL in our institution, each patient initially received the same standard treatment including i.v. Dexamethasone 0.15 mg/kg + i.v. Glycerol (10%, 500 ml) for 10 days, followed by additional 10 days of oral steroid tapering. In case of treatment failure, salvage therapy with intratympanic steroid injections (Dexamethasone 24 mg/ml, five times in 3 weeks) and/or hyperbaric oxygen therapy (10 sessions in 2 weeks) and/or fibrinogen/LDL-apheresis (one session) were attempted. Only selected cases with highest WML scores according to the Fazekas scale were sent to neurological evaluation and only a subset of them started antiplatelet treatment accordingly.

### 2.10. Follow up and subgroups of patients

Pure tone, impedance and speech audiometries were scheduled according to the following timeline from admission to assess hearing recovery: 15 days, 3, 6, and 12. Audiometries performed on admission and at 6 months were considered to assess the final hearing improvement. Hearing recovery was considered either “complete” (if PTA returned within 10 dB HL of the unaffected ear, with WRS >50%), “partial” (PTA improvement >10 dB HL without returning within 10 dB HL of the unaffected ear, with WRS >50%) or “no recovery” (either PTA improvement <10 dB HL or greater improvement with a WRS ≤ 50%) (1). The amount of PTA recovered was calculated by the difference between presenting PTA (PTApre) and PTA at 6-month follow up (PTApost) for the four most impaired contiguous frequencies on the affected ear as follows: PTApre – PTApost. Conversely, the mean percentage of hearing recovery was calculated comparing the amount of PTA recovered to the difference between presenting PTA on the affected ear and the PTA for the corresponding affected frequencies on the contralateral ear (PTAcontra) as follows: (PTApre – PTApost) / (PTApre – PTAcontra) × 100. Even patients' symptoms were followed up over the 12 months from admission and three different subgroups were identified according to the clinical course. In case of lack of vestibular symptoms both on admission and throughout the whole follow up, the patient was assigned to the “SSNHL no vertigo” subgroup. Conversely, in case vestibular symptoms only occurred simultaneously with SSNHL, the patient was assigned to the “SSNHL + vertigo” subgroup. Finally, in case of fluctuating/relapsing HL, later onset of vertigo or recurrent vestibular symptoms consistent with “definite” or “probable” MD, according to the 2015 guidelines (72), the patient was considered as “MD.” Finally, some patterns of vestibular impairment were identified as likely due to a “vascular” pathomechanism, based on the vascular supply to the inner ear and clinical descriptions (57–59, 61–63, 65–68). The following patterns were considered “vascular”:

- High-frequency SSNHL associated with an impairment for cVEMPs and PSC VOR-gain, with or without concurrent abnormal oVEMPs, consistent with a selective ischemia in the territory mainly supplied by the vestibulo-cochlear artery (VCA).
- Severe or profound SSNHL associated with an impairment for cVEMPs and PSC VOR-gain, with or without concurrent abnormal oVEMPs, consistent with a selective ischemia in the territory mainly supplied by the common-cochlear artery (CCA).
- Severe or profound SSNHL associated with both otolith and SC impairment, consistent with a selective ischemia in the territory supplied by the IAA.

### 2.11. Statistical analysis

Statistical analysis was performed with IBM SPSS Statistics, version 20.0 for Windows (IBM Corp.; NY, USA). Categorical variables were presented as percentages. Quantitative variables were checked for normal distribution using both Kolmogorov–Smirnov and Shapiro-Wilk tests. Continuously distributed variables were described by mean ± 1 standard deviation or by median, interquartile range, and range. Pearson Chi-square test or Fischer exact test were used for categorical comparisons. A logistic regression model was used to evaluate the effects of prognostic factors on hearing recovery. The Kruskal–Wallis one-way analysis of variance (ANOVA) was used to compare median values of instrumental variables and *post-hoc* analysis was applied to compensate for multiple comparisons. Statistical significance was presented as *p*-value and assumed when a null hypothesis could be rejected at *p* < 0.05.

## 3. Results

### 3.1. Demographics and presenting clinical/instrumental findings of overall cohort

During the period examined, 147 patients were hospitalized. Sixty-one patients were excluded from the study either due to incomplete audio-vestibular assessment (18 patients), due to previous inner ear disease (40 patients) or known SSNHL etiology (three patients: vestibular schwannoma in one case, acoustic trauma in another case and multiple sclerosis in an additional case). Finally, 86 patients with complete instrumental assessment met the inclusion criteria for idiopathic SSNHL on admission and were included in the analysis: 49 were males (57%) and 37 females (43%), with a median age of 55.7 years ± 14.4 (range age 22–84 years old). None of them present with clinical or radiological signs of CNS involvement and none showed screening test consistent with active viral infection (HSV, HZV, CMV). SSNHL occurred on the right side in 41.9% of cases (36/86) and 45/86 (52.3%) patients complained of vestibular symptoms on admission. The average time between symptoms onset and the beginning of treatment was 14.4 ± 10.7 days (range 1–30 days). Hearing loss was mild in 12.8% of cases (11/86), moderate in 47.7% (41/86), severe in 18.6% (16/86) and profound/anacusis in 20.9% of cases (18/86). Hearing loss was down-sloping in 25.6% of cases (22/86), involved low-frequency tones in 24.4% (21/86) and was flat in 50% (43/86).

**Figure 1:**
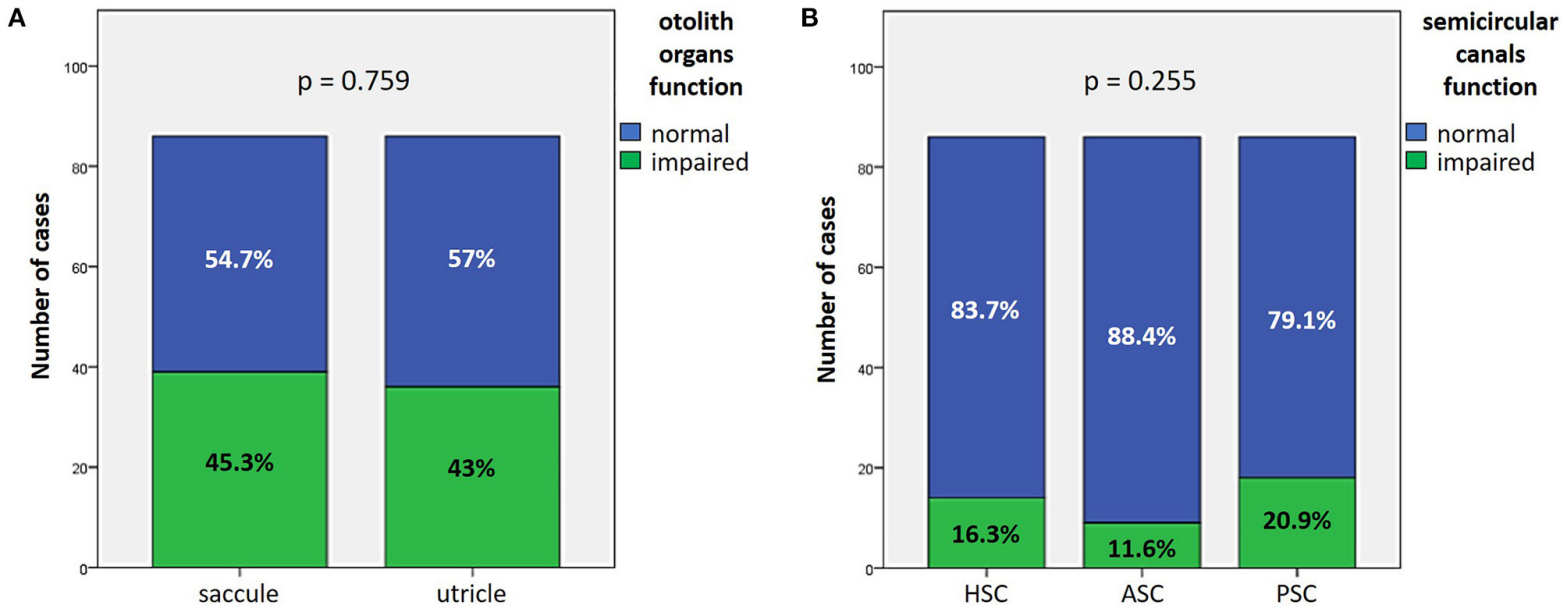
Bar plots showing the number of cases among overall cohort of 86 cases exhibiting different instrumental findings. **(A)** Normal or impaired function of otolith receptors. **(B)** Normal or impaired function of semicircular canals. Relative percentages are reported in each column and *p*-values of categorical comparisons are reported. ASC, anterior semicircular canal; HSC, horizontal semicircular canal; PSC, posterior semicircular canal.

Otolith receptors were involved in similar percentage of cases as saccular function was impaired in 45.3% of cases (39/86), while utricular function in 43% (37/86) according to cervical and ocular VEMPs measurements, respectively (*p* = 0.759; Figure 1A). Similarly, HSC function was altered on vHIT in 16.3% (14/86), ASC in 11.6% (10/86) and PSC in 20.9% of cases (18/86) with no statistically significant difference (*p* = 0.255; Figure 1B).

### 3.2. Presenting clinical/instrumental findings in different subgroups

Once divided the overall cohort according to presenting symptoms and clinical course, 25/86 patients (29.1%) fit the “SSNHL no vertigo” subgroup, 27/86 patients (31.4%) were assigned to the “SSNHL + vertigo” subgroup and the remaining 34 patients (39.5%) in “MD.” Compete clinical and instrumental data of overall patients from each subgroup can be in found in Supplementary Tables a–c. Subgroups did not significantly differ in terms of patients' age (*p* = 0.092), time between symptoms onset and beginning of treatment (*p* = 0.979) and number of cardiovascular risk factors (*p* = 0.394).

#### 3.2.1. Cochlear function

**Figure 2:**
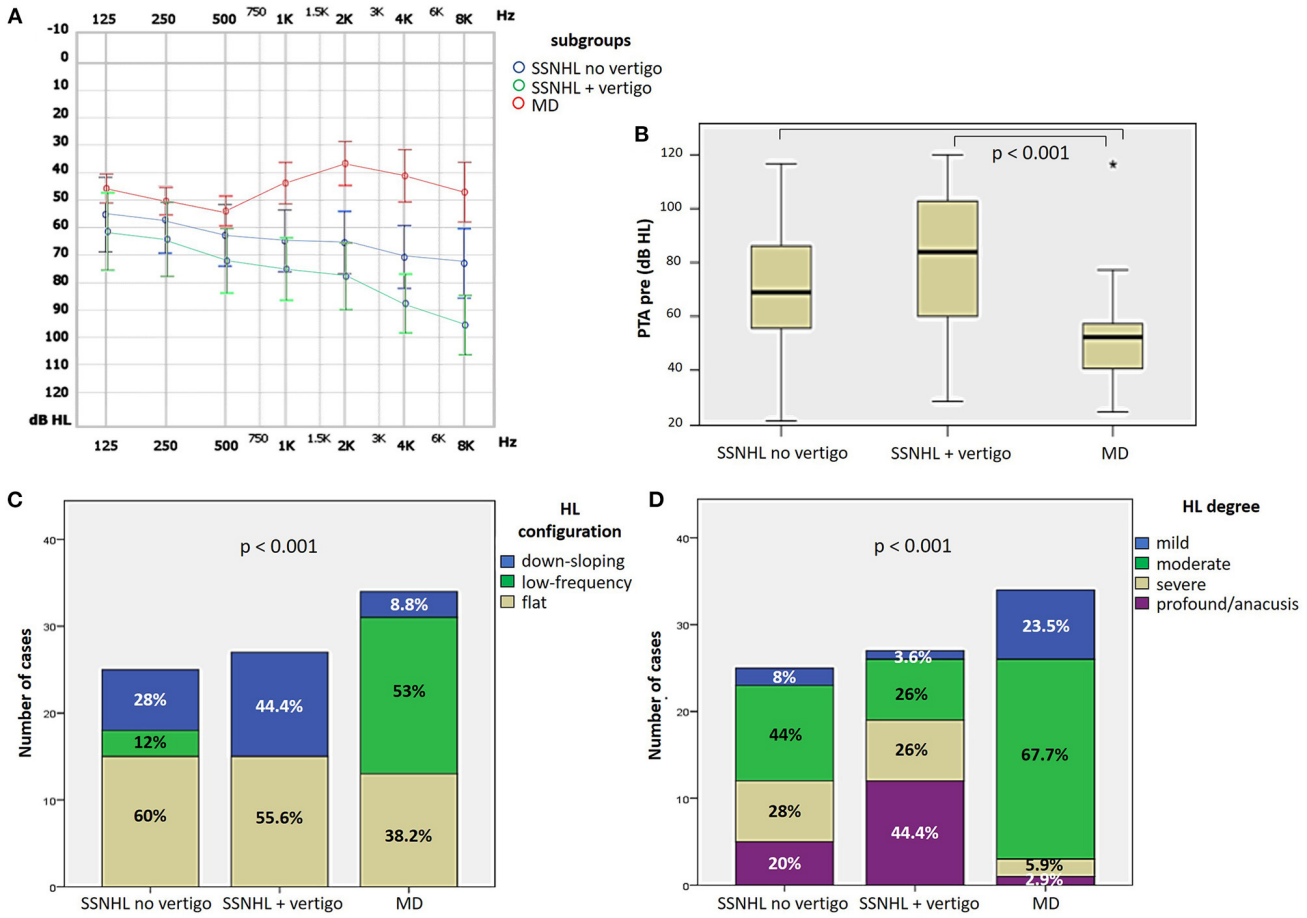
Presenting audiometric features of the affected ear of the overall cohort, divided in subgroups based on clinical course. **(A)** Audiogram. **(B)** Box plot correlating median values of PTA among subgroups. Statistically significant differences at the ANOVA test are shown. **(C, D)** Bar plots showing the number of cases presenting with different HL configurations **(C)** and different HL degree **(D)** among subgroups. Relative percentages are reported in each column and *p*-values of categorical comparisons are reported. HL, hearing loss; MD, Menière's disease; PTA pre, presenting pure tone average; SSNHL, sudden sensorineural hearing loss.

Presenting cochlear function was more affected in “SSNHL + vertigo” patients and less impaired in “MD” (*p* < 0.05; Figures 2A, B). HL configuration and degree with corresponding statistically significant different distribution among subgroups are reported in Figures 2C, D. In particular, subjects without vestibular symptoms mainly presented either with down-sloping or flat HL and with moderate to severe HL. Similarly, presenting HL of patients with “SSNHL + vertigo” was either flat or down-sloping; nevertheless, HL degree was higher than the other subgroups, being mild in only 3.6% and profound/anacusis in 44.4% of cases. On the contrary, “MD” patients mainly presented either with low-frequency or flat HL with mean PTA mostly ≤ 70 dB.

#### 3.2.2. Otolith function

**Figure 3:**
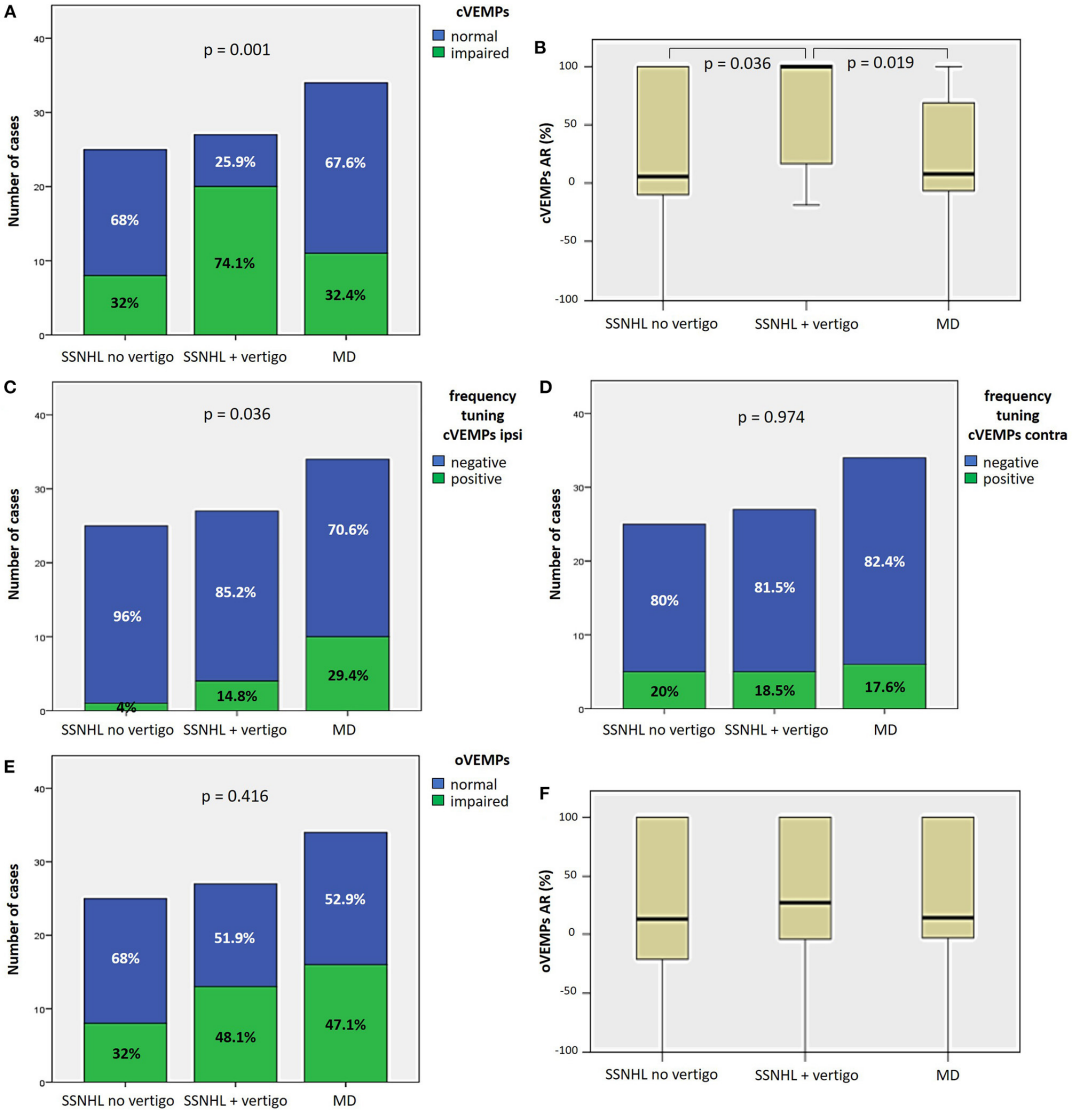
Different distribution of saccular **(A–D)** and utricular dysfunction **(E, F)**, as assessed by c and oVEMPs, respectively, among different subgroups based on clinical course. **(A)** Bar plot showing the distribution of impaired saccular function of the side affected by SSNHL among different subgroups. Relative percentages are reported in each column and *p*-value of categorical comparisons is reported. **(B)** Box plot correlating median values of cVEMPs AR among subgroups. Statistically significant differences at the ANOVA test are shown. Bar plots showing the distribution of positive frequency tuning of cVEMPs of the side affected by SSNHL **(C)** and of the contralesional side **(D)** among different subgroups. Relative percentages are reported in each column and *p*-values of categorical comparisons are reported. **(E)** Bar plot showing the distribution of impaired utricular function of the side affected by SSNHL among different subgroups. Relative percentages are reported in each column and *p*-value of categorical comparisons is reported. **(F)** Box plot correlating median values of oVEMPs AR among subgroups. No statistically significant differences at the ANOVA test are shown. AR, asymmetry ratio; contra, contralesional ear; cVEMPS, cervical vestibular-evoked myogenic potentials; ipsi, affected ear; MD, Menière's disease; oVEMPs, ocular vestibular-evoked myogenic potentials; SSNHL, sudden sensorineural hearing loss.

Saccular impairment as assessed through cVEMPs was more frequently registered in the group of “SSNHL + vertigo” patients (20/27, 74.1%, *p* = 0.001; Figure 3A) and, similarly, the extent of abnormal AR for cVEMPs was greater in the same subgroup compared to the others (*p* < 0.05; Figure 3B). While a positive frequency tuning for ipsilesional cVEMPs was more frequently found in MD patients (10/34, 29.4%, *p* = 0.036), there were no significantly different distribution of positive frequency tuning among subgroups in the contralateral ear (*p* = 0.974; Figures 3C, D). Conversely, neither the prevalence of utricular impairment as assessed by oVEMPs (*p* = 0.416) nor the AR of oVEMPs amplitudes (*p* = 0.516) significantly differed among subgroups (Figures 3E, F).

#### 3.2.3. Canal function

**Figure 4:**
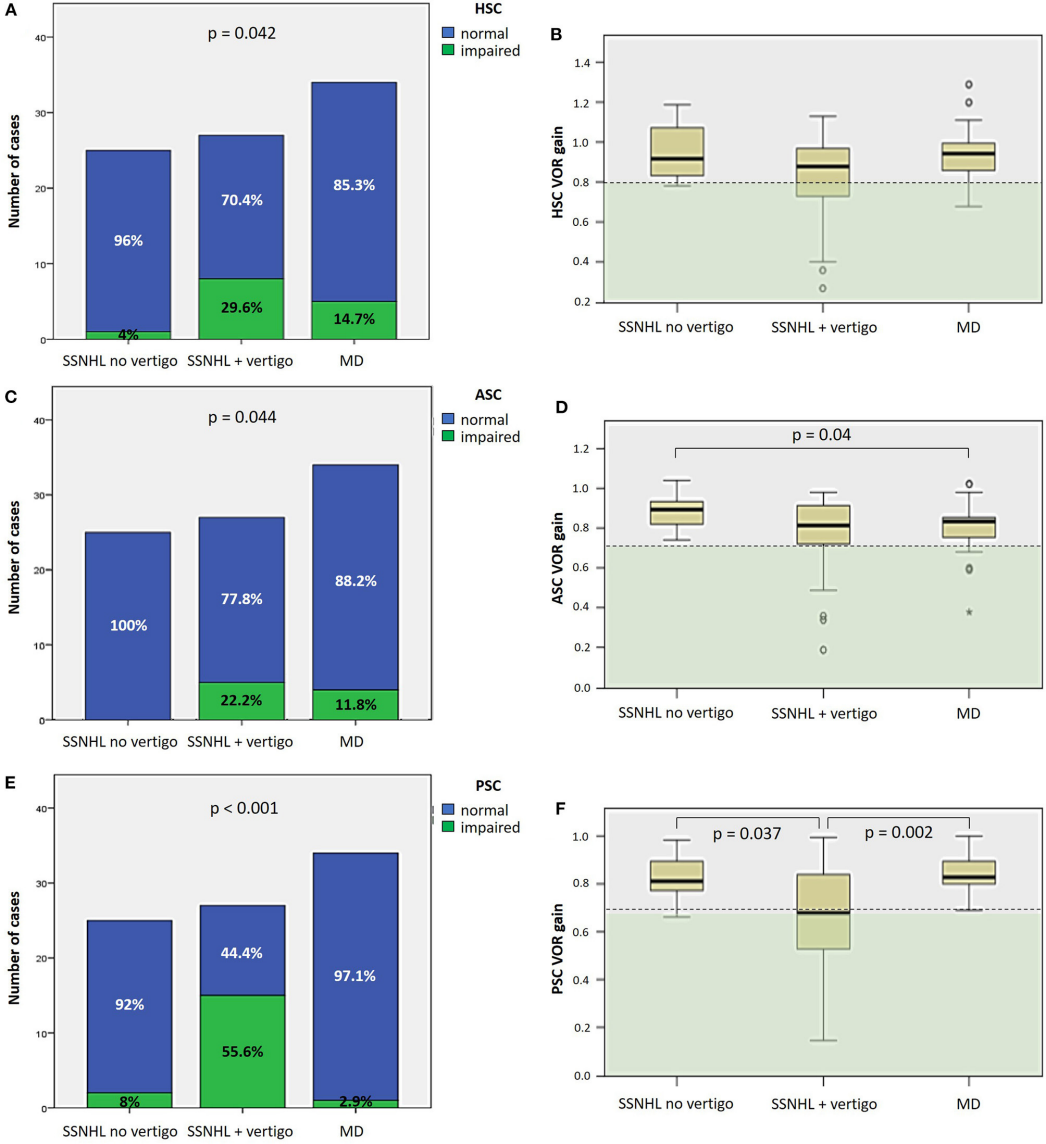
Different distribution of semicircular canal dysfunction, as assessed by vHIT, among different subgroups based on clinical course. **(A, C, E)** Bar plots showing the distribution of impaired HSC **(A)**, ASC **(C)** and PSC function **(E)** of the side affected by SSNHL among different subgroups. Relative percentages are reported in each column and *p*-values of categorical comparisons are reported. **(B, D, F)** Box plots correlating median values of HSC **(B)**, ASC **(D)**, and PSC VOR gain **(F)** among subgroups. Statistically significant differences at the ANOVA test are shown. ASC, anterior semicircular canal; HSC, horizontal semicircular canal; MD, Menière's disease; PSC, posterior semicircular canal; SSNHL, sudden sensorineural hearing loss; VOR, vestibulo-ocular reflex; vHIT, video-head impulse test.

As for SC function, the percentage of cases with HSC impairment on vHIT significantly differed among subgroups, being more frequently impaired in “SSNHL + vertigo” (8/27, 29.6%) and in “MD” patients (5/34, 14.7%; *p* = 0.042), albeit without significant different mean VOR-gain values (Figures 4A, B). The percentage of subjects with ASC impairment significantly differed among subgroups (*p* = 0.044) as all patients without vestibular symptoms (25/25, 100%) exhibited spared ASC function, and “MD” patients presented with significantly lower ASC VOR-gain values than patients without vestibular symptoms (*p* = 0.04; Figures 4C, D). Finally, PSC function was significantly more affected in the “SSNHL + vertigo” subgroups compared to the others in terms of both percentage of cases presenting with PSC impairment (15/27, 55.6%, *p* < 0.001) and mean VOR-gain values on vHIT (Figures 4E, F).

#### 3.2.4. Vestibular lesion patterns

**Figure 5:**
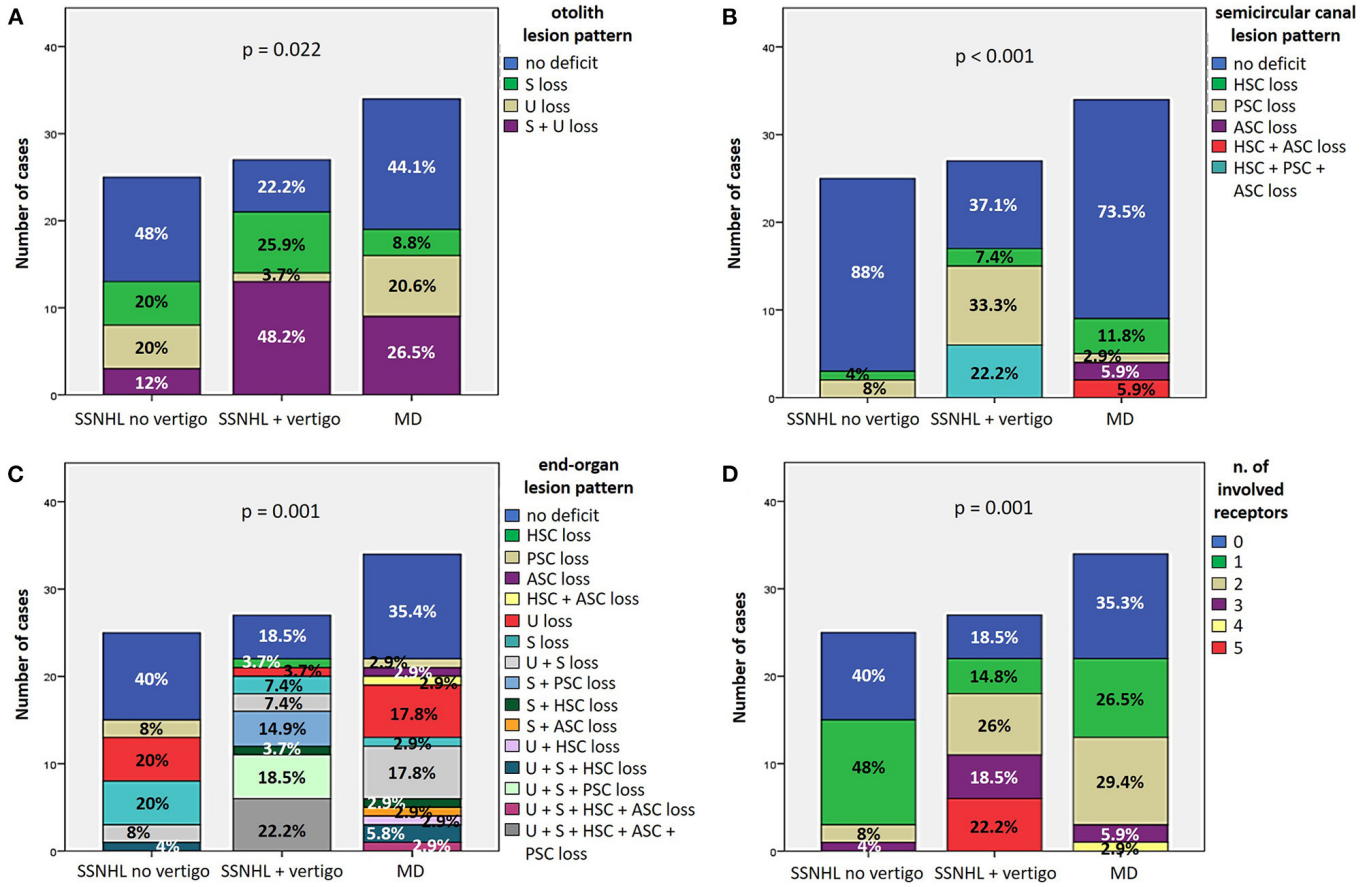
**(A**, **B)** Bar plots showing the different distribution of otolith **(A)** and semicircular canal **(B)** lesion patterns among different subgroups based on clinical course. Relative percentages are reported in each column and *p*-values of categorical comparisons are reported. **(C, D)** Bar plots showing the different distribution of end-organ lesion patterns **(C)** and the amount of impaired vestibular receptors **(D)** among different subgroups. Relative percentages are reported in each column and *p*-value of categorical comparisons is reported. ASC, anterior semicircular canal; HSC, horizontal semicircular canal; MD, Menière's disease; PSC, posterior semicircular canal; S, sacculus; SSNHL, sudden sensorineural hearing loss; U, utriculus.

Patterns of otolith and SC impairment were analyzed and reported in Figures 5A, B, respectively, with corresponding statistically significant difference in distribution among subgroups. Overall distribution of otolith lesion patterns significantly differed among subgroups (*p* = 0.022), as the “SSNHL + vertigo” subgroup exhibited a smaller percentage of patients without otolith involvement (6/27, 22.2%) than other subgroups and, on the other hand, a higher percentage of cases with both saccular and utricular damage (13/27, 48.2%; Figure 5A). Conversely, while in the vast majority of “SSNHL no vertigo” and “MD” patients overall SC function was spared (22/25, 88% and 25/34, 73.5%, respectively), 62.9% of patients with “SSNHL + vertigo” (17/27) presented with at least 1 SC damaged. In particular, patients without vertigo only presented at most with a selective impairment involving either the HSC (1/25, 4%) or the PSC (2/25, 8%), whereas isolated PSC impairment represented the most frequent SC lesion pattern detected among “SSNHL + vertigo” patients (9/27, 33.3%). Conversely, the subset of “MD” patients presenting with canal dysfunction (9/34, 26.5%) most frequently exhibited a selective HSC impairment on vHIT (4/34, 11.8%) and represented the only cohort displaying a selective ASC damage (2/34, 5.9%). Furthermore, while simultaneous impairment of HSC and ASC could be only detected in “MD” patients (2/34, 5.9%), only the “SSNHL + vertigo” subgroup exhibited a SC impairment involving all the three SC (6/27, 22.2%; Figure 5B). Furthermore, the distribution within subgroups of the end organ lesion patterns and overall number of impaired vestibular receptors with the corresponding value of statistical significance (*p* = 0.001) is reported in Figures 5C, D, respectively. While the vast majority of patients included in the “SSNHL no vertigo” subgroup exhibited at most 1 impaired vestibular sensor (22/25, 88%), 91.2% of “MD” patients (31/34) exhibited at most 2 lesioned vestibular end-organs, whereas 66.7% of “SSNHL + vertigo” patients (18/27) displayed at least two involved sensors. Similarly, while more than three affected sensors could be detected in none in the “SSNHL no vertigo” subgroup, 2.9% (1/34) of “MD” patient presented with four impaired sensors and 22.2% (6/27) of patients with “SSNHL + vertigo” showed a complete vestibular damage. Noteworthy, no functional impairment of vestibular end-organs could be detected in five patients included in this latter subgroup (18.5%).

#### 3.2.5. Video-Frenzel findings

**Figure 6:**
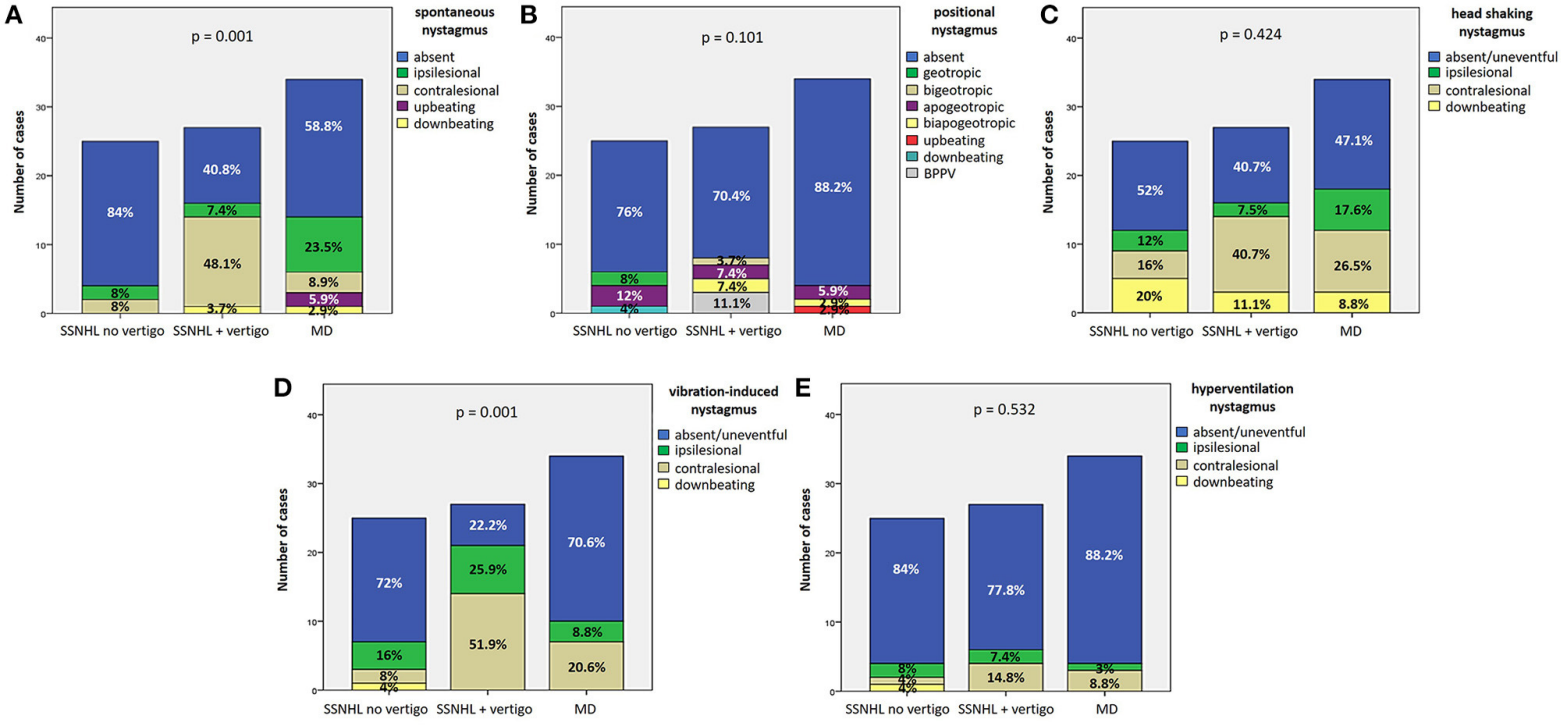
Bar plots showing the different distribution of the features of spontaneous **(A)**, positional **(B)**, head shaking **(C)**, vibration-induced **(D)**, and hyperventilation nystagmus **(E)** among different subgroups based on clinical course. Relative percentages are reported in each column and *p*-values of categorical comparisons are reported. MD, Menière's disease; SSNHL, sudden sensorineural hearing loss.

As for spontaneous/positional nystagmus on video-Frenzel examination, none of the patients showed gaze-evoked nystagmus or other oculomotor signs attributable to CNS involvement. SN could be detected in 39.5% (34/86) of patients (Table 1), while PN could be found in only 20.9% of cases (18/86), presenting as horizontal apogeotropic PN in 11.6% (10/86), horizontal geotropic PN in 3.5% (3/86), paroxysmal upbeating PN consistent with ipsilesional PSC-BPPV in three cases (3.5%), persistent downbeating PN in one case and persistent upbeating PN in an additional case. While the distribution of SN greatly differed among subgroups (*p* = 0.001), a similar minority of patients among the 3 subgroups exhibited PN (*p* = 0.101; Figures 6A, B). In particular, in “SSNHL no vertigo” subgroup, while most patients presented neither with SN (21/25, 84%) nor with PN (19/25, 76%), a subset of patients exhibited either contralesional (i.e., paretic; 2/25, 8%) or ipsilesional SN (2/25, 8%) despite denying vestibular symptoms, and PN could be found in additional 6/25 patients (24%). On the other hand, the majority of “SSNHL + vertigo” patients presented with SN (16/27, 59.2%), being contralesional in most cases (13/27, 48.1%) while ipsilesional in only two cases and downbeating in 1; all patients with PSC-BPPV were included in this subgroup accounting for 11.1% of cases (3/27). Conversely, less than half of “MD” patients (14/34, 41.2%) presented with heterogenous types of SN, exhibiting mainly ipsilesional (i.e., either irritative or recovery) SN in 23.5% of cases (8/34), paretic SN in 8.9% (3/34), upbeating SN in 5.9% (2/34) and downbeating SN in 2.9% (1/34). The vast majority of them showed no PN (30/34, 88.2%), while only three (8.8%) presented with apogeotropic PN and only one case with persistent upbeating PN. Noteworthy, both upbeating SN and persistent upbeating PN could only be detected in “MD” patients. Details of remaining oculomotor findings on video-Frenzel examination for overall cohort including HSN, VIN and HVN are summarized in Table 1. HSN did not significantly differ among subgroups (*p* = 0.424), despite only in the “SSNHL no vertigo” subgroup head shakings neither elicited any detectable nystagmus nor significantly modified ongoing SN in the majority of cases (13/25, 52%). Conversely, contralesional HSN was more frequent among “SSNHL + vertigo” patients (11/27, 40.7%) while ipsilesional HSN was more frequently found among “MD” (6/34, 17.6%; Figure 6C). On the other hand, VIN significantly differed among subgroups (*p* = 0.001). In particular, whereas 25.9% (7/27) of “SSNHL + vertigo” subjects developed ipsilesional VIN, it was mainly contralesional in this subgroup (14/27, 51.9%), while it was absent in the majority of cases for the other subgroups (Figure 6D). Even HVN features and distribution did not significantly differ among subgroups (*p* = 0.532), as in the vast majority of cases hyperventilation test neither resulted in detectable eye movements nor modified ongoing SN (Figure 6E). Interestingly, only one case in the “SSNHL no vertigo” subgroup exhibited downbeating nystagmus after skull vibration and hyperventilation tests.

**Table 1:** Video-Frenzel findings of the overall cohort of 86 patients.

	**Absent (*n*, %)**	**Contra (*n*, %)**	**Ipsi (*n*, %)**	**Downbeating (*n*, %)**	**Upbeating (*n*, %)**
SN	52/86 (60.5%)	18/86 (20.9%)	12/86 (14%)	2/86 (2.3%)	2/86 (2.3%)
HSN	53/86 (61.6%)	15/86 (17.4%)	8/86 (9.4%)	10/86 (11.6%)	0/86 (0%)
VIN	63/86 (73.2%)	12/86 (14%)	10/86 (11.6%)	1/86 (1.2%)	0/86 (0%)
HVN	75/86 (87.2%)	5/86 (5.8%)	5/86 (5.8%)	1/86 (1.2%)	0/86 (0%)

Contra, contralesional; HSN, head shaking nystagmus, HVN, hyperventilation nystagmus; ipsi, ipsilesional; SN, spontaneous nystagmus; VIN, vibration-induced nystagmus.

### 3.3. Hearing recovery

**Figure 7:**
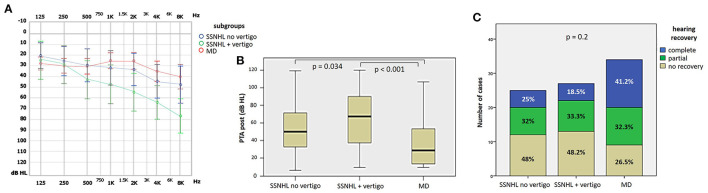
Final audiometric features at 6-month follow up of the affected ear of the overall cohort, divided in subgroups based on clinical course. **(A)** Audiogram. **(B)** Box plot correlating median values of PTA among subgroups. Statistically significant differences at the ANOVA test are shown. **(C)** Bar plot showing the different distribution of hearing recovery among different subgroups. Relative percentages are reported in each column and *p*-value of categorical comparisons is reported. MD, Menière's disease; PTA post, pure tone average at 6-month follow up; SSNHL, sudden sensorineural hearing loss.

**Figure 8:**
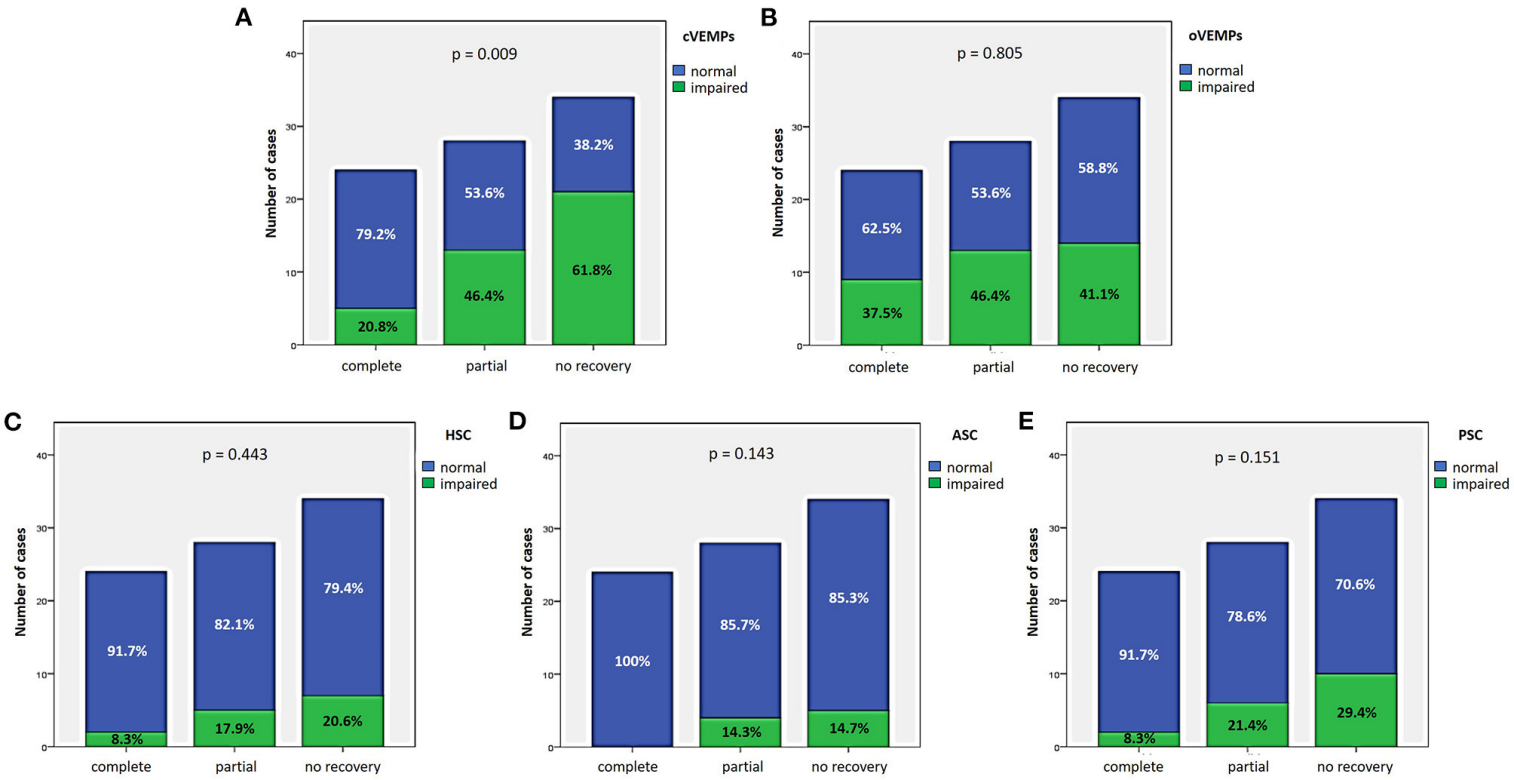
Bar plots showing the different distribution of otolith **(A, B)** and semicircular canal impairment **(C–E)** ipsilesionally to SSNHL, as measured by cVEMPs, oVEMPs, and vHIT, respectively, once divided the cohort according to hearing recovery. Relative percentages are reported in each column and *p*-value of categorical comparisons are reported. ASC, anterior semicircular canal; cVEMPs, cervical vestibular-evoked myogenic potentials; HSC, horizontal semicircular canal; oVEMPs, ocular vestibular-evoked myogenic potentials; PSC, posterior semicircular canal; SSNHL, sudden sensorineural hearing loss; vHIT, video-head impulse test.

**Figure 9:**
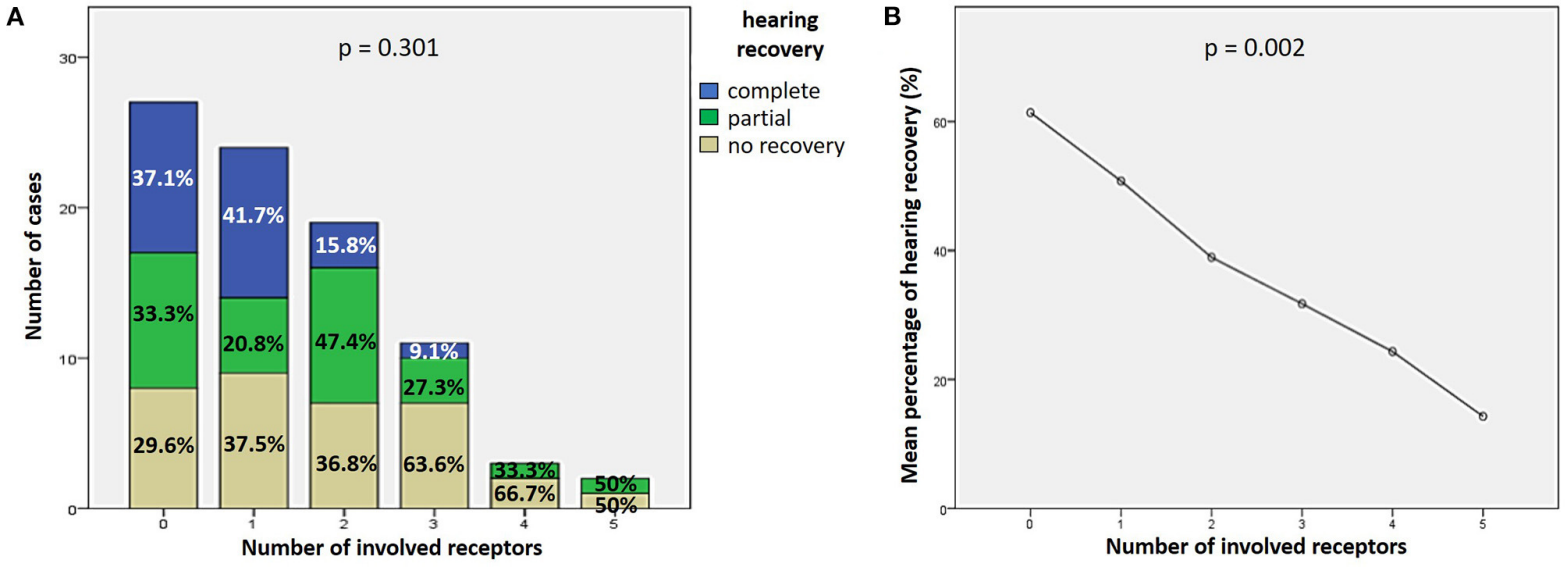
Correlation between the number of impaired vestibular receptors ipsilesionally to SSNHL and hearing recovery. **(A)** Bar plots showing the different distribution of hearing recovery among the study cohort divided according to the number of affected sensors. **(B)** Correlation trend between the number of impaired vestibular receptors and mean percentage of hearing recovery.

**Figure 10:**
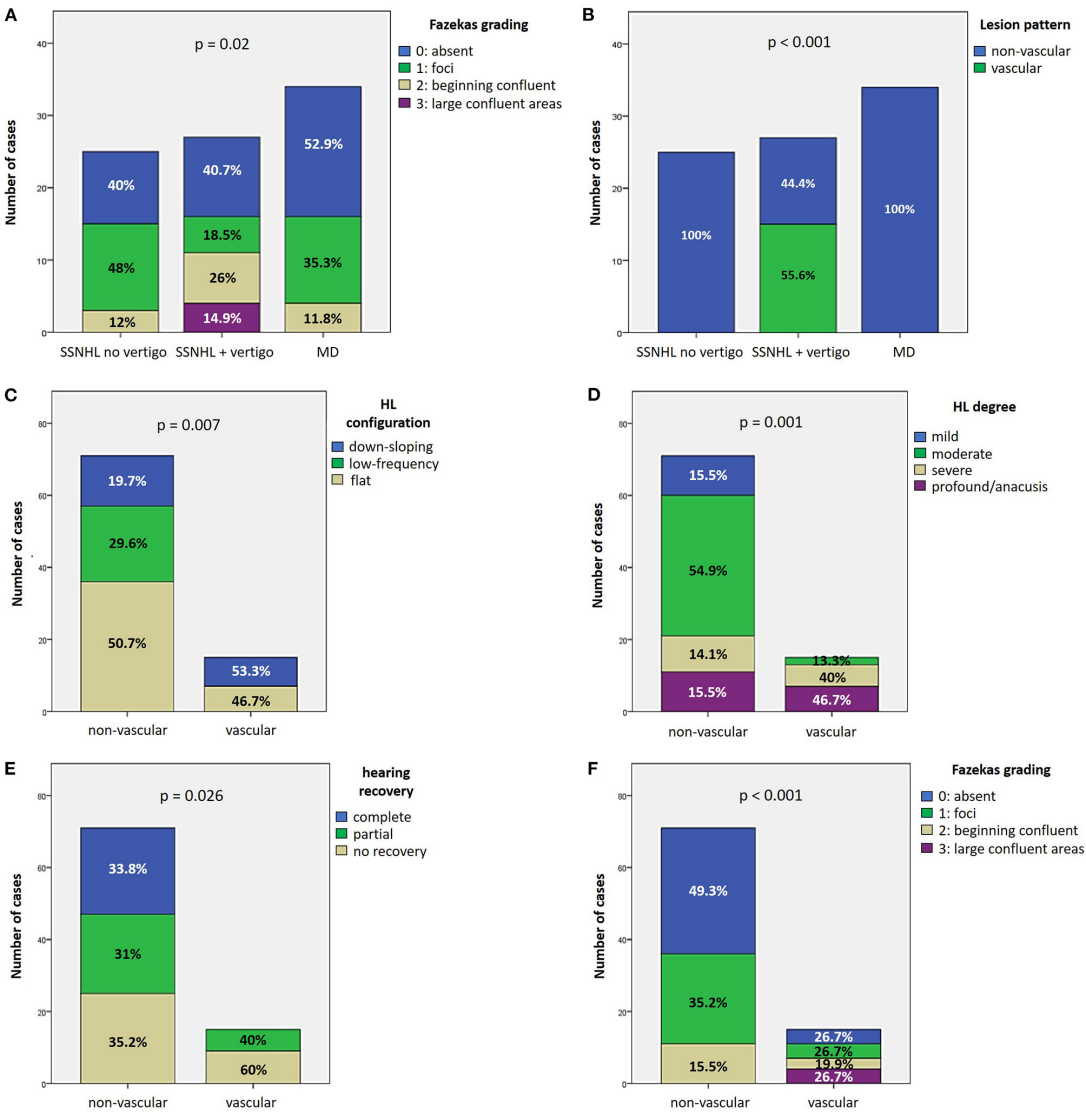
Vascular patterns. **(A, B)** Bar plots showing the different distribution of Fazekas grading of WML on MRI **(A)** and instrumental lesion patterns consistent with a vascular lesion **(B)** among the study cohort divided according to the clinical course. **(C–F)** Bar plots showing the different distribution of HL configuration **(C)**, HL degree **(D)**, hearing recovery **(E)**, and Fazekas grading of WML on MRI **(F)** among the study cohort divided according to the instrumental lesion patterns consistent with a vascular lesion. Relative percentages are reported in each column and *p*-value of categorical comparisons are reported. HL, hearing loss; MD, Menière's disease; SSNHL, sudden sensorineural hearing loss; WML, white matter lesions.

27.9% of cases (24/86) exhibited complete hearing restoration, 32.6% (28/86) developed partial recovery and 39.5% (34/86) had not hearing recovery, according to current guidelines (1). “MD” patients resulted in the best hearing function at 6-month follow up after treatment compared to the other subgroups (*p* < 0.05), as reported in Figures 7A, B. Even though “MD” exhibited a greater proportion of complete recovery (14/34 of cases, 41.2%) compared to other subgroups, no statistically relevant difference could be found in the distribution of different degree of hearing recovery among subgroups (Figure 7C). Once divided the overall cohort according to hearing outcome in “complete,” “partial” and “no recovery,” only saccular dysfunction resulted to be significantly associated with a poor prognosis (*p* = 0.009; Figure 8A). Even though the function of all sensors was more frequently impaired in cases with no hearing recovery and spared in cases with complete auditory restoration, nor utricular neither SC impairment significantly correlated with hearing outcome (*p* > 0.05; Figures 8B–E). The extent of inner lesion was significantly associated with hearing recovery; in particular, while the distribution of different types of hearing recovery did not significantly differ among patients divided according to the number of impaired vestibular receptors (*p* = 0.301; Figure 9A), there was a statistically significant negative correlation trend between the number of damaged sensors and the mean percentage of hearing recovery (*p* = 0.002; Figure 9B). The distribution of Fazekas score significantly differed among subgroups (*p* = 0.02; Figure 10A). Notably, while 88% (22/25) of subjects in “SSNHL no vertigo” subgroup and 88.2% (30/34) of “MD” patients exhibited at most grade 1 WML, “SSNHL + vertigo” patients exhibited the highest amount of WML, as 85.2% (23/27) of cases presented with at most grade 2 WML and all 4 patients with grade 3 lesions were included in this subgroup.

### 3.4. Vascular patterns: Association with presenting hearing loss and hearing recovery

**Figure 11:**
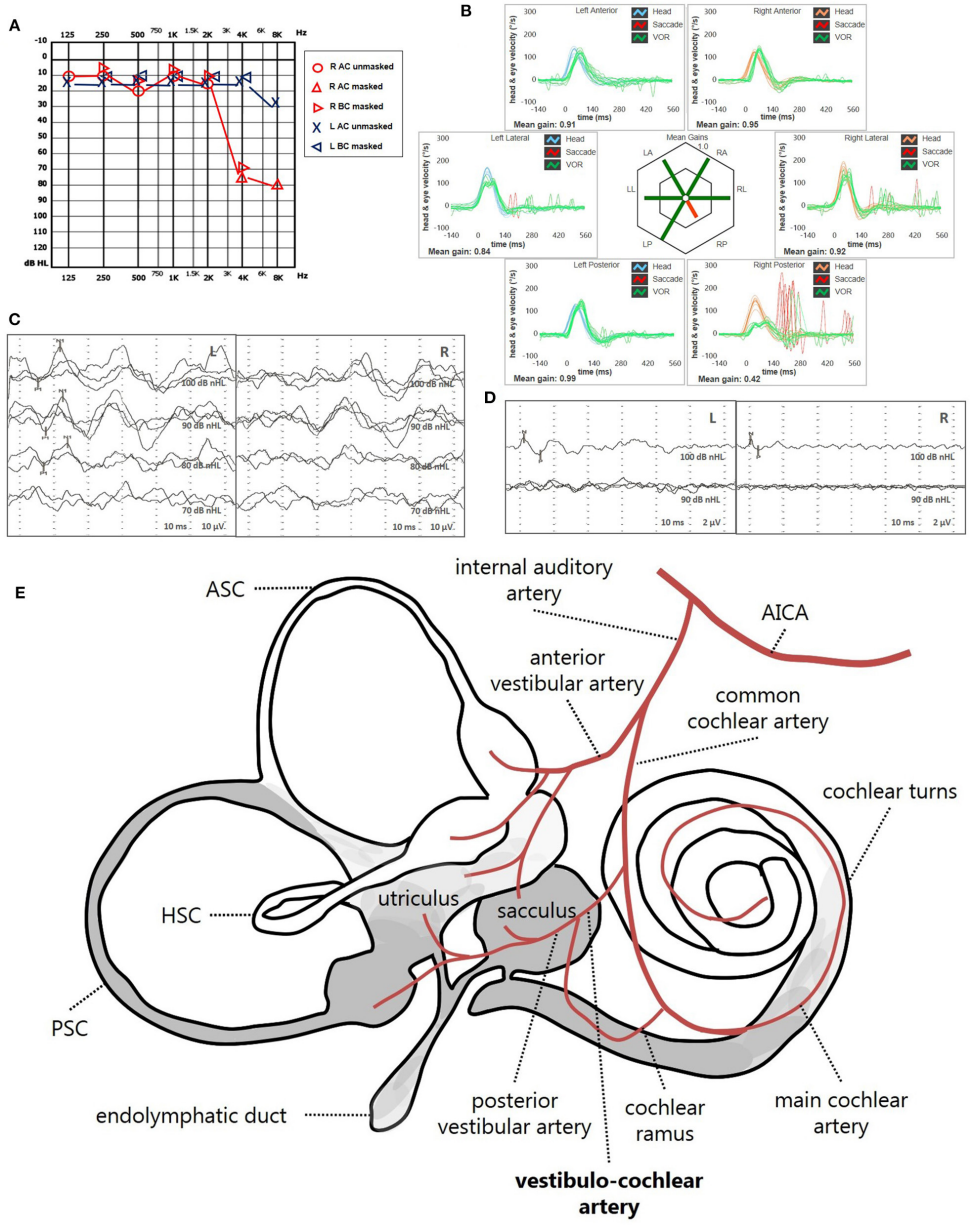
Presenting scenario of patients #42 with an instrumental lesion pattern consistent with an ischemic damage in the territory mainly supplied by the right VCA (see also the Supplementary Video 1). **(A)** Pure-tone audiometry exhibiting high-frequency sensorineural HL on the right side. **(B)** vHIT showing a selective VOR-gain impairment for the right PSC (0.42) with overt saccades. **(C)** cVEMPs revealing absent potentials on the right side and normal responses on the left side (AR = 100%). **(D)** oVEMPs with potentials on both sides with an amplitude asymmetry within normality range (L > R, AR = 29%). **(E)** Schematic representation of the vascular supply of the inner ear, highlighting the assumed ischemic pathomechanism [modified from Schuknecht (63)]. Labyrinthine receptors mainly supplied by the VCA are represented in gray. AC, air-conduction; AICA, anterior-inferior cerebellar artery; ASC, anterior semicircular canal; BC, bone-conduction; cVEMPs, cervical vestibular-evoked myogenic potentials; HSC, horizontal semicircular canal; L, left; LA, left anterior; LL, left lateral; LP, left posterior; oVEMPs, ocular vestibular-evoked myogenic potentials; PSC, posterior semicircular canal; R, right; RA, right anterior; RL, right lateral; RP, right posterior; VCA, vestibulo-cochlear artery; vHIT, video-head impulse test; VOR, vestibulo-ocular reflex.

**Figure 12:**
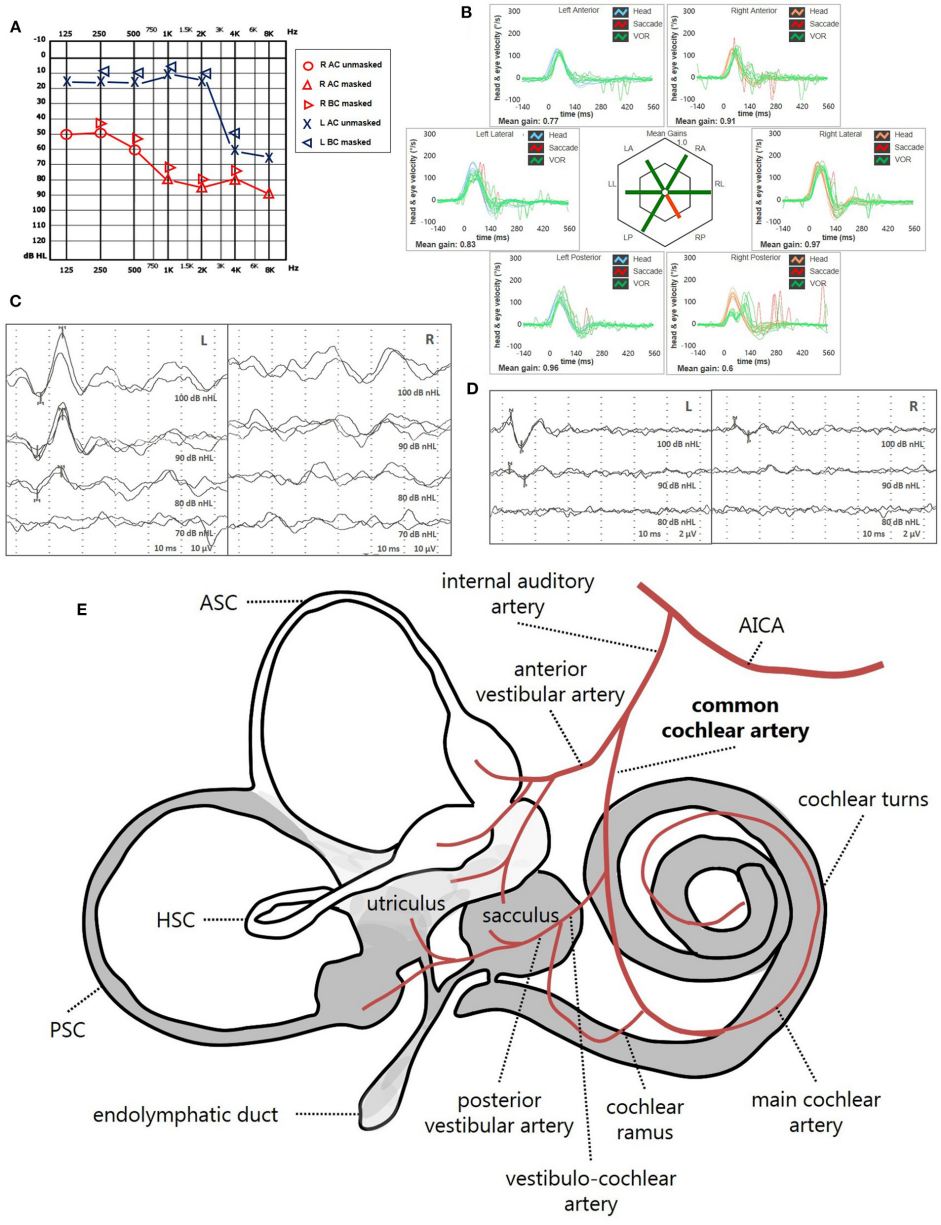
Presenting scenario of patients #64 with an instrumental lesion pattern consistent with an ischemic damage in the territory mainly supplied by the right CAA (see also the Supplementary Video 2). **(A)** Pure-tone audiometry exhibiting severe down-sloping sensorineural HL on the right side. **(B)** vHIT showing a selective VOR-gain impairment for the right PSC (0.6) with both overt and covert saccades. **(C)** cVEMPs revealing absent potentials on the right side and normal responses on the left side (AR = 100%). **(D)** oVEMPs with potentials on both sides with an abnormal amplitude asymmetry (L > R, AR = 33%). **(E)** Labyrinthine receptors mainly supplied by the CCA are represented in gray. AC, air-conduction; AICA, anterior-inferior cerebellar artery; ASC, anterior semicircular canal; BC, bone-conduction; CCA, common-cochlear artery; cVEMPs, cervical vestibular-evoked myogenic potentials; HSC, horizontal semicircular canal; L, left; LA, left anterior; LL, left lateral; LP, left posterior; oVEMPs, ocular vestibular-evoked myogenic potentials; PSC, posterior semicircular canal; R, right; RA, right anterior; RL, right lateral; RP, right posterior; vHIT, video-head impulse test; VOR, vestibulo-ocular reflex.

**Figure 13:**
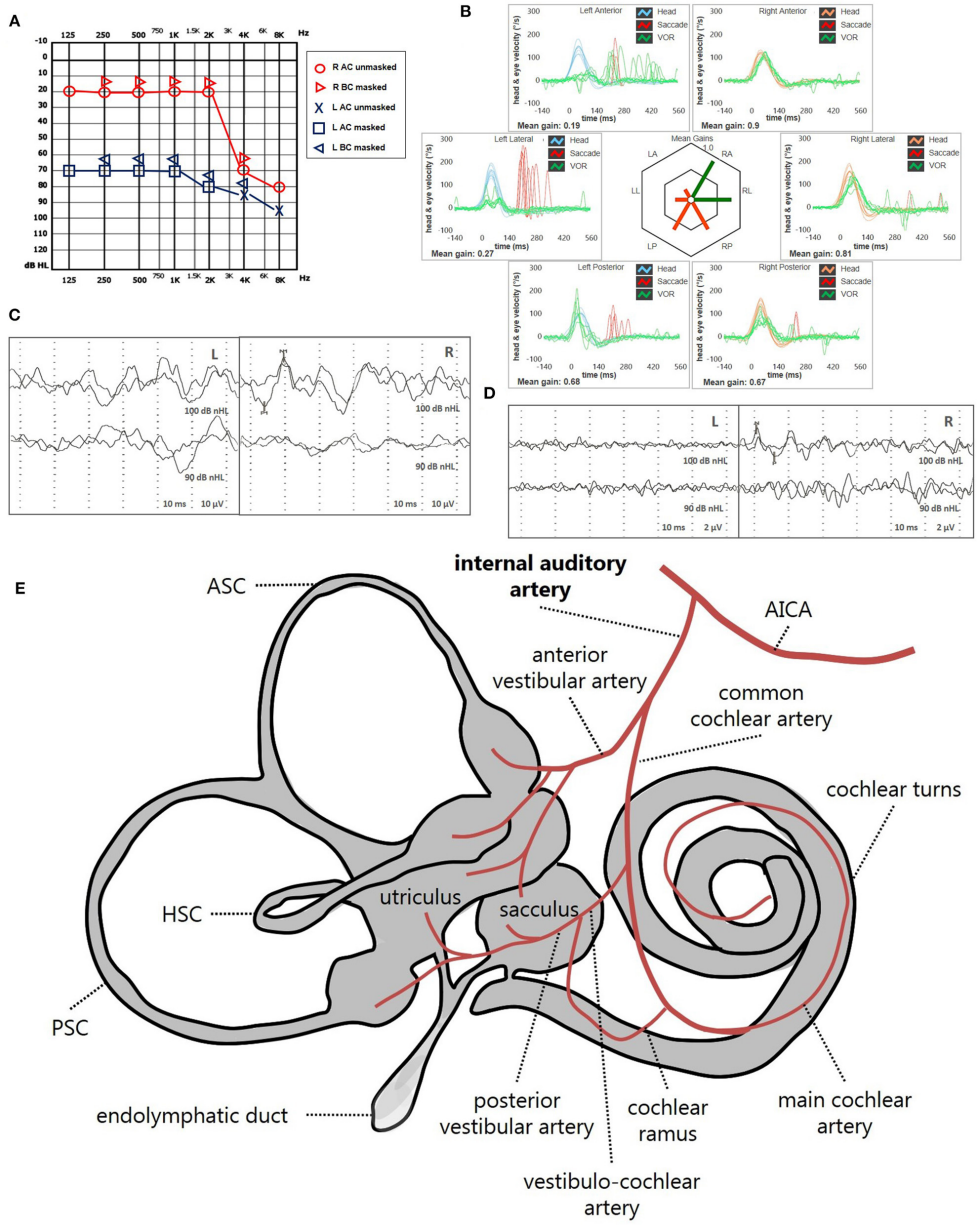
Presenting scenario of patients #59 with an instrumental lesion pattern consistent with an ischemic damage in the territory mainly supplied by the left IAA (see also the Supplementary Video 3). **(A)** Pure-tone audiometry exhibiting severe flat sensorineural HL on the left side. **(B)** vHIT showing VOR-gain impairment for all the left SCs with mainly overt saccades. cVEMPs **(C)** and oVEMPs **(D)** revealing absent potentials on the left side and normal responses on the right (AR = 100%). **(E)** Labyrinthine receptors mainly supplied by the IAA are represented in gray. AC, air-conduction; AICA, anterior-inferior cerebellar artery; ASC, anterior semicircular canal; BC, bone-conduction; CCA, common-cochlear artery; cVEMPs, cervical vestibular-evoked myogenic potentials; HSC, horizontal semicircular canal; L, left; LA, left anterior; LL, left lateral; LP, left posterior; oVEMPs, ocular vestibular-evoked myogenic potentials; PSC, posterior semicircular canal; SC, semicircular canal; R, right; RA, right anterior; RL, right lateral; RP, right posterior; vHIT, video-head impulse test; VOR, vestibulo-ocular reflex.

Instrumental lesion patterns most likely consistent with an ischemic lesion of the inner ear were identified in 17.4% (15/86) of patients. Notably, two patients exhibited instrumental dysfunctions consistent with a VCA ischemic lesion (Figure 11, Supplementary Video 1), seven presented with clinical findings most likely due to a vascular lesion in the territory supplied by the CCA (Figure 12, Supplementary Video 2) and remaining six fulfilled diagnostic criteria of IAA occlusion (Figure 13, Supplementary Video 3). Interestingly, all of them belonged to the “SSNHL + vertigo” subgroup, accounting for more than half (15/27, 55.6%) of patients herein included (Figure 10B). Even HL configuration and degree significantly differed between patients presenting either with “vascular” or “non-vascular” lesion patterns. In particular, while all cases presenting with low-frequency SSNHL (21/71) were included only in the “non-vascular” subgroup, accounting for 29.6% of cases (*p* = 0.007), patients in “vascular” subgroups only presented either with down-sloping (8/15, 53.3%) or flat HL (7/15, 46.7%; Figure 10C). On the other hand, in more than half of patients presenting with “non-vascular” lesion patterns (39/71, 54.9%) a moderate SSNHL could be detected, and all cases with mild SSNHL (11/71) were included in this category, accounting for 15.5% of cases. Conversely, “vascular” cases never presented with mild SSNHL but mostly with either severe HL (6/15, 40%) or profound HL/anacusis (7/15, 46.7%; *p* = 0.001; Figure 10D). Additionally, different lesion patterns were found to have a significantly different impact on hearing recovery, as a full hearing restitution could only be obtained in patients exhibiting “non-vascular” lesion patterns (24/71, 33.8%), while all “vascular” cases could only reach either a partial (6/15, 40%) or no hearing recovery (9/15, 60%; *p* = 0.026; Figure 10E). Finally, we could confirm a clearly different distribution of MRI findings between patients either with “vascular” or “non-vascular” patterns of instrumental impairment (*p* < 0.001; Figure 10F). In fact, while almost half of “non-vascular” patients were lacking in WML on MRI (35/71, 49.3%), the vast majority of “vascular” patients (11/15, 73.3%) exhibited WML, including overall four patients with grade 3 WML according to Fazekas score, accounting for 26.7% of cases herein included.

## 4. Discussion

Predicting hearing outcome in SSNHL is still challenging, as well as detecting the precise pathomechanism accounting for symptoms and clinical findings. SSNHL could be associated with vestibular lesions since cochlear turns, otolith receptors and SCs share the same embryological origin and vascular supply. Moreover, besides being in close anatomical proximity, vestibular and cochlear hair cells provide afferents running through the internal acoustic canal in different but closely attached branches of the VIII cranial nerve. In fact, the VIII cranial nerve is composed by the cochlear nerve (CN) and the VN: while the latter receives fibers from both the superior and inferior VN, CN collects afferents from the entire spiral ganglion. In turn, the superior VN is composed of the lateral and anterior ampullary nerves and the utricular nerve, whereas the inferior VN is composed of the posterior ampullary nerve and the saccular nerve (63). On the other hand, the inner ear is supplied by the IAA, which branches from the anterior-inferior cerebellar artery (AICA) and divides into two main terminal branches: the anterior vestibular artery and CCA. Whereas, the first mostly supplies the utricle, the upper part of the saccule and both ASC and HSC, the latter mainly serves the cochlea, saccule, the lower part of the utricle and PSC. The VCA branches from the CCA and divides into the posterior vestibular artery, which provides blood supply to saccule and PSC, and the cochlear ramus that serves the basal turn of the cochlea. The main cochlear artery supplies the rest of the cochlear neuroepithelium. (63, 65–68). While selective damages to inner ear sensors (either cochlear or vestibular) could be attributed with certainty neither to neural nor to vascular damage, being possible both neuritis involving portions of VN/CN afferents and ischemia involving vestibular/cochlear end-organs, when SSNHL accompanies acute vestibular symptoms the lesion site should be searched within the labyrinth itself and an ischemic damage should be always suspected (73, 74). For example, while a selective lesion involving the sole PSC and saccule should orient toward a neural damage involving the inferior VN (54, 55), in the case of associated SSNHL a vascular damage should be considered, as the shared susceptibility of these structures may reflect the common vascular supply of the pars inferior of the labyrinth given by the CCA (29, 32, 57–59, 61, 62). Clinicians should be aware of this eventuality as it has been demonstrated how peripheral ischemic lesion may precede a posterior fossa stroke (69, 70). Even though neuritis involving both CN and VN have been described, likely due to a spreading of the damage through the anastomosis between the CN and the IVN (75–77), as well as an isolated ischemia involving the end-organs giving afferents into the superior VN or the inferior VN, likely due to an occlusion either of the anterior or posterior vestibular artery, respectively (73, 78, 79), the most easily assumable pathomechanism accounting for an acute cochleovestibular damage seems to be ischemia, in particular in the case an extensive instrumental assessment detects a precise lesion pattern overlapping the territories supplied by the IAA or its main branches. The lesion site of vestibular disorders in SSNHL with vertigo appeared to be within the labyrinth even on the basis of galvanic-VEMP findings (38). Configuration of HL might also provide useful informations in terms of pathophysiology. In fact, while low-frequency SSNHL is thought to be caused by the damage of the apical turns of the cochlea, an impairment of the higher frequencies should identify a damage of the basal turns. The latter cochlear portion is anatomically closer to vestibular organs, in particular the saccular macula, therefore it is easy to understand why high-frequency SSNHL tend to be more related to vestibular damages (27, 32, 39, 46). Nevertheless, some researches showed that also patients with low-frequency SSNHL tend to develop vertigo attacks (18, 19, 22). This data suggests that the anatomical position of the labyrinthine sensors and their topographical organization based on innervation and vascular supply may not fully explain the whole spectrum of symptoms in patients with SSNHL. In some of these cases, chemical and/or density alterations of perilymph and/or endolymph should be considered among pathomechanisms, since cochlear and vestibular partitions share the same inner ear fluids and are interconnected by surrounding membranes. Although its exact pathophysiology remains unclear, MD has been recognized as an idiopathic syndrome related to EH resulting in episodic vertigo attacks, fluctuating hearing loss, tinnitus, and aural fullness (18, 19, 22). Therefore, when SSNHL and vestibular symptoms coexist, in particular with an impairment of low tones on audiometry, EH and related disorders should always be investigated. Nonetheless, MD is a fluctuating disease, accounting for transient vestibulo-cochlear dysfunctions, and EH likely represents a common stage of different abnormalities presenting with similar symptoms. All these factors might account for the extreme heterogeneity of instrumental data related to MD, preventing the identification of specific patterns of audio-vestibular dysfunction for MD. In fact, different types of oculomotor findings, SC involvement and otolith alterations have been described based on the stage of the disease and the extent of EH (16, 20, 80–87). Although 3T MRI with delayed acquisition following Gadolinium infusion has demonstrated to detect EH in most patients with MD (13, 16, 17, 21, 24), physiological confirmation of EH is still useful for the diagnosis of MD in clinical setting. Due to the anatomical alterations that the hydropic inner ear develops over time, the coexistence of a reduced caloric response and normal HSC-VOR gin at the vHIT has been considered a hallmark of MD (17, 88). The tuning property test seems to represent another interesting and peculiar data supporting EH in MD, as these patients tend to show 1-kHz dominant VEMPs responses in comparison with VEMPs responses to 500 Hz due to saccular hydrops, while healthy subjects show 500-Hz dominant VEMPs responses (14, 15).

Given the aforementioned basis, it is easy to understand why vestibular assessment in SSNHL and its role in clinical presentation and hearing outcome have been widely explored in the literature. Most studies evaluated the vestibular function mainly through caloric testing, cVEMPs and oVEMPs, reporting contrasting data, in particular regarding its prognostic role. While most studies found that both abnormal caloric responses and, in general, the extent of inner ear damage correlate with the severity of HL and with poor recovery, there is no consensus on which vestibular end-organ is mostly involved and whether canal or otolith impairment is more closely related to worse hearing recovery (26, 28, 30, 31, 34–36, 38–50). One of the possible factors accounting for these discrepancies could be that comparing caloric test (which assess the low-acceleration response of the sole HSC) with VEMPs (which measures otolith reflexes in the high-frequency domain) does not seem to represent the most proper way to test vestibular end-organs. In fact, the higher vulnerability to various disorders of caloric responses might lead to biased topographic data on inner ear damage, as well as the different recovery behavior of different hair-cell populations might affect prognostic data. In recent years, since when the vHIT replaced the scleral search-coil system in the measurements of ampullary reflexes in the high frequency domain (52), the vestibular test battery in clinical setting has been extended to the evaluation of vertical SCs (29, 32, 34, 37, 49, 50, 57). According to the vast majority of the most recent investigations, PSC represents the most frequently involved SC in SSNHL, in particular when acute vertigo accompanies hearing symptoms (29, 32, 34, 37, 50, 57). Moreover, PSC hypofunction on vHIT appears to be a specific prognostic factor for incomplete hearing recovery, while impaired HSC VOR-gain has been related to mild and moderate SSNHL (34, 49). Oculomotor findings, including SN, PN and other evoked nystagmus, have also been investigated in SSNHL. According to the literature, the detection of SN seems to be related to PSC impairment, while it correlates with a higher incidence of recurrent HL in cases with low-frequency SSNHL. Additionally, BPPV seems to be associated with deeper HL and worse recovery rate (28, 50, 89). In particular, if the occurrence of BPPV ipsilesionally to SSNHL has been easily explained either with arterial occlusions or selective multiple vascular or neural involvement causing HL and otoconial release from the utricle, a change in density and viscosity of the endolymph by inner ear hypoperfusion or light/heavy debris attached to the cupula has been suggested as a possible cause of buoyancy mechanisms generating geotropic/apogeotropic PN, respectively (80–93). Similar biochemical alterations in the inner ear fluid exerting initially excitotoxic effect and then inhibition to vestibular afferents, along with central reorganization due to vestibular compensation, have also been assumed to explain spontaneous conversion of SN in SSNHL (94). Similar nystagmus behavior (both spontaneous and positional) has been registered both in the ictal and inter-ictal stage of MD, raising the question of whether the patients with SSNHL associated either to spontaneous direction-changing or positional direction-changing nystagmus should be considered as MD (80, 87, 94, 95).

In the present study, we aimed to evaluate the vestibular function in patients with SSNHL with or without vertigo and to explore its role in the prediction of hearing recovery and in the early detection of the underlying pathomechanisms. We hypothesized that the assessment of the vestibular function by means of vHIT, cVEMPs, and oVEMPs and the evaluation of SN, PN, HSN, VIN, and HVN through Video-Frenzel goggles might provide prognostic information and might suggest meaningful information for understanding the pathophysiology of the disease. We therefore divided the patients according to the clinical presentation and the evolution of symptoms, defining three different profiles: isolated SSNHL without vertigo (“SSNHL no vertigo”), SSNHL with acute vertigo (“SSNHL + vertigo”) and MD (“MD”). Since it is well-known how patient's age, vascular comorbidities and time between onset of symptoms and treatment are considered the most influencing factors on hearing outcome (5, 6, 26, 96, 97), we preliminarily verified that those factors did not show statistically significant differences among subgroups in order to avoid bias during statistical analysis. As for vestibular function in the overall cohort, our data confirmed previous studies and systematic review stating that otolith organs were the most susceptible to damage in SSNHL (33, 35, 38), even if we did not find significant differences between otolith organs and among SCs (*p* > 0.05). Once exploring the differences among subgroups, hearing was more impaired in “SSNHL + vertigo” patients, who exhibited either down-sloping or flat-type audiogram, and was less impaired in “MD,” where low frequencies were mostly impaired (*p* < 0.001). These results are consistent with previously reported data highlighting how HL is worse, in particular the highest frequencies, in patients with vertigo compared to non-vertigo (27, 32, 37, 51). Nevertheless, even a part of patients with MD exhibited down-sloping and flat HL, in accordance with other reports on “MD-like” patients (20, 21). Among otolith receptors, cVEMPs were more affected in patients with “SSNHL + vertigo” than other subgroups (*p* = 0.027) and, similarly, this population exhibited the highest rate of combined impairment of both cVEMPs and oVEMPs and showed the lowest percentage of isolated utricular deficit (*p* = 0.022), confirming how vestibular end-organs close to the cochlea tend to be preferentially affected in patients with vertigo (46). When exploring the tuning frequency of cVEMPs, in accordance with literature, we found that the rate of 1-kHz dominant VEMPs responses were higher in “MD” patients ipsilesionally to HL (*p* = 0.036) (14, 15). Nonetheless, only 29.4% of ears with MD exhibited a positive frequency tuning, while a small percentage of contralesional ears and of patients not fitting diagnostic criteria for MD presented with the same behavior. These discrepancies might be explained considering that all patients in our study cohort presented with the first episode of audio-vestibular symptoms, implying that MD patients herein investigated were at the earliest stage of the disease. Additionally, we gathered subjects with “definite” and “probable” MD in the same group; according to studies where potential differences between individuals with “definite” and “probable” MD were explored, Authors found that the dominant frequency shifted toward 1 kHz only in individuals with “definite” MD (14, 15). On the other hand, the visualization of EH through 3T Gadolinium-enhanced brain MRI in 65% of asymptomatic contralateral ears could account for the presence of a tuning shift toward higher stimulation frequencies also in non-MD subjects (13, 17). Another factor accounting for the presence of a positive tuning curve in non-MD patients might be the evidence of EH even in a subgroup of patients with idiopathic SSNHL, likely as a secondary reaction following a damage of inner ear sensors (21, 24). Interestingly, 14 patients exhibited an AR ≤ -33% either for cVEMPs or oVEMPs. This peculiar lesion pattern due to an enhanced otolith reflexes of the affected ear has been described in the early stage of MD patients, likely due to an increased endolymphatic stiffness induced by an hydropic state of the vestibular compartment (81, 98). In line with this assumption, six of these patients were “MD,” while five belonged to the “SSNHL no vertigo” subgroup and only three to the “SSNHL + vertigo” subgroup. Since 11 of these patients presented either with low-frequency or flat SSNHL, 10 of them with mild to moderate HL, while none of them exhibited a WML score >1, it is possible to assume that most of these cases developed EH in the affected ear. Although these patients exhibited neither a reduced VEMPs threshold nor conductive HL, and two of them developed a VOR-gain impairment for ipsilesional SCs (PSC in one case and ASC in the other), temporal bone high-resolution CT scan was scheduled to exclude a third window disorder. In fact, it has been demonstrated how SC dehiscence might account for low-frequency HL, enhanced VEMPs responses and functional impairment of the affected SC at the vHIT on the same ear (99). As for vHIT data, the “SSNHL + vertigo” subgroup exhibited a higher prevalence of PSC loss compared to the others (*p* < 0.001), confirming previous results on SCs function in patients with sudden deafness and vestibular symptoms (29, 32, 34, 37, 49, 50, 57). Conversely, patients with “SSNHL no vertigo” exhibited the lowest rate of SC impairment even for HSC and ASC (*p* < 0.05). Only MD developed a SC lesion pattern consistent with isolated ASC involvement and HSC + ASC damage, while only patients with vertigo presented with all SCs impaired. Considering overall vestibular lesion patterns, patients with “SSNHL no vertigo” mainly presented either with no deficit, with isolated utricular loss or with isolated saccular loss, while “SSNHL + vertigo” subjects mainly developed either functional loss for overall receptors or PSC + saccular loss with/without utricular impairment. Conversely, despite MD exhibited the most heterogeneous findings, the most frequent patterns included either no sensor impairment, isolated utricular impairment or hypofunction of both otolith receptors (*p* = 0.001). Overall number of involved sensors was lowest for patients without vertigo and highest for those with vertigo (*p* = 0.001). As already reported in the literature, we noted that not all patients showing some degree of vestibular impairment complained of vertigo (in particular, 52% of cases without vertigo developed otolith dysfunctions) and, on the contrary, patients with vestibular symptoms did not always have vestibular function abnormalities on instrumental test (18.5%) (34, 37, 49). Either previous asymptomatic dysfunctions or damages with a slowly progressive onset leading to central compensation mechanisms could likely account for the lack of vestibular symptoms in subjects with altered vestibular data. Conversely, normal instrumental tests for patients with vestibular complaints strengthen the significance of data collected from temporal bones of patients with SSNHL where no direct relationship between the presence of vertigo and damage to the vestibular apparatus could be found. In these cases, it has been hypothesized that vertigo might be due to the transmission of biochemical changes in the inner ear fluid between cochlear and vestibular partition or that the damage could involve the extracellular superstructure (100, 101). It should also be considered the possibility that a fast functional recovery might occur in some damaged receptor, as the time span between the onset of symptoms and the evaluation was ≥3 days for all these patients.

SN behaved differently in the three subgroups, being absent in most patients without vertigo and MD, and mostly contralesional in the “SSNHL + vertigo” subgroup. Furthermore, MD subgroup exhibited higher rate of ipsilesional SN compared to others and was the sole subgroup exhibiting upbeating SN, while both “SSNHL + vertigo” and “MD” subgroups presented small percentages of downbeating SN (*p* = 0.001). These findings are in accordance with previous investigation (32, 50, 61, 80, 87, 94). Conversely, the three subgroups exhibited similar small rates of PN with some exceptions; in fact, only one patient without vertigo developed downbeating PN and only one MD patient exhibited upbeating PN. As reported in other studies (28, 32, 50), also three patients from the “SSNHL + vertigo” subgroup developed ipsilesional PSC-BPPV that resolved in all cases after appropriate canalith repositioning maneuvers. Noteworthy, only one patient exhibited utricular and HSC/ASC impaired as expected from an ischemic involvement of the anterior vestibular artery or from a neural damage involving the superior VN leading to an otolith dislodgment from the utricle. Irrespective of the underlying cause, this discrepancy can be explained with a faster recovery of these structures during the time between the onset of symptoms and clinical testing. Conversely, since the utricle receives a dual vascular supply, it might be assumed that otolith dislodgement might result from an ischemic lesion involving the territory supplied by a branch of the CCA. Additionally, two of the three patients who developed PSC-BPPV exhibited reduced VOR gain for the PSC involved. This apparently incongruent finding might be explained assuming a functional dissociation between “transient” and “sustained” vestibular system encoding angular accelerations (102). In particular, paroxysmal PN despite SC impairment on vHIT might imply a selective damage for type I hair-cells and irregular canal afferents (measured by vHIT) sparing the activity of type II hair-cells and regular fibers encoding cupular displacements which generates nystagmus (103–105). On the other hand, it may be assumed a different recovery time for damaged end-organs/afferents following acute labyrinthine injury, resulting in faster restoration for low-frequency responses compared to higher-frequency VOR (106, 107). Nevertheless, a possible role of the residual function of irregular afferents in the genesis of paroxysmal PN could not be excluded, as these patients did not develop a complete PSC loss on vHIT. Another interesting finding closely related to the aforementioned assumption is that only 50% (17/34) of patients presenting with SN exhibited a canal dysfunction at the vHIT, whereas at least an otolith receptor was impaired (mainly the utricle) in 11 cases. This atypical pattern was most frequently observed in “MD” subgroup, where 10/14 patients with SN did not exhibit canal impairment and the utricle was involved in five cases. Though this data might strengthen the hypothesis that SN might be generated by a selective utricular damage (108), it could also be assumed that in most of these patients an underlying EH could more likely dampen the activity of type II hair cells and regular afferents encoding low-frequency inputs, resulting in SN and in the classical dissociation between vHIT (spared) and caloric responses (abnormal) (17, 88). Unfortunatey, we could not confirm these hypotheses since caloric irrigations were not performed in our study. Another interesting finding is that nystagmus was detected in 72% of cases without vertigo, raising the hypothesis that some of them might develop in future a clinical picture consistent with MD.

Though HSN did not provide useful information to distinguish the three different subgroups, it is worthwhile to highlight that it elicited downbeat nystagmus in a small percentage of subjects. This finding, along with horizontal SN with no HSC VOR-gain impairment on vHIT or the sole vertical SN, could have been misdiagnosed as central disorders (2, 3, 109). Conversely, it has already been already demonstrated how vertical components perverted HSN in MD and acute vertigo with SSNHL might be related either to asymmetrically impaired vertical SCs (PSC more than ASC) or misorientation of the velocity storage mechanism (55, 62, 82). On the other hand, VIN behaved highly differently among subgroups. Given that it is known to represent a sensitive and simple clinical test for detecting peripheral vestibular asymmetry, it makes sense that it was mainly elicited in “SSNHL + vertigo” patients with contralesional direction (*p* = 0.001). Even though this group exhibited mainly PSC hypofunction, no downbeating VIN was detect as expected from an asymmetrical activation of the opposed paired vertical SCs, but rather it was mostly horizontal as reported in the literature (77). This data might be explained assuming either an extent of ischemic damage to the only HSC hair-cells encoding low-frequency signals (thus not detectable with the vHIT) or by a previous wider damage involving the HSC, where only type-I hair-cells have recovered over time (61). The relatively lower percentage of VIN in MD patients in our cohort (29.4%) compared to literature might be explained recalling that all our patients were at the earliest stage of the disease and that SVIN is correlated with the severity of caloric hypofunction, likely mild in early-stage MD (110). Finally, rates of HVN were extremely low in all subgroups, strengthening the assumption that most pathomechanisms underlying SSNHL involve inner ear structures rather than VN/CN fibers (77, 111). In fact, HVN has been reported to occur in the case of alterations in the neuronal excitability in the vestibular system. In particular, ipsilesional HVN might be due to a transitory improvement of axonal conduction in partially demyelinated nerve fibers resulting from an increase in cerebrospinal fluid pH by hyperventilation, while brain vasoconstriction resulting from high-velocity deep breaths might affect the velocity storage mechanism which had restored the resting neuronal excitability after peripheral vestibular deficit, accounting for contralesional HVN (112). Nevertheless, it is well-known how hyperventilation can induce ipsilesional nystagmus in vestibular neuritis in the acute stage. The significance of our findings needs to be scaled back since a stratification of the behavior of HVN according to the time span from the onset of symptoms and evaluation was not pursued.

As for hearing recovery, the final HL was less impaired in “MD” and more impaired in “SSNHL + vertigo” subgroup (*p* < 0.001). Nevertheless, rates of hearing recovery did not significantly differ among subgroups. These data are in agreement with previous data on the better recovery low-tones SSNHL (19, 22, 42) and in that SSNHL patients with vertigo have a worse prognosis, while there is no difference in whole rate of hearing recovery between the different groups (27, 28, 37). Our data are also consistent with the literature on the correlations between vestibular function and hearing recovery. In particular, we confirmed that an incomplete hearing recovery is significantly associated with saccular impairment (*p* = 0.009) and with the number of impaired vestibular receptors (*p* = 0.002) (31, 33, 35, 37–39, 41, 42, 46, 51). Finally, we aimed to investigate whether “vascular” lesion pattern correlated with the worst hearing impairment at presentation and poorest prognosis. According to studies on inner ear vascularization and clinical reports, three specific lesion patterns seem to be assumable as “vascular” with cautious confidence, being otherwise hardly explainable (57–59, 61–63, 65–68):

- High-frequency SSNHL associated with an impairment for PSC VOR-gain, cVEMPs and with or without concurrent abnormal oVEMPs, due to a lesion involving the cochlear basal turn, the PSC, the saccule and possibly a part of the utricle, consistent with a selective occlusion of the VCA (Figure 11).
- Severe or profound SSNHL associated with an impairment for PSC VOR-gain, cVEMPs and with or without concurrent abnormal oVEMPs, due to a lesion involving more extensively the cochlear turns, the PSC, the saccule and possibly a part of the utricle, consistent with a selective ischemia in the territory mainly supplied by the CCA (Figure 12).
- Severe or profound SSNHL associated with an impairment of cVEMPs, oVEMPs and all three SC-VOR-gain, due to a complete inner ear damage, consistent with an IAA infarct (Figure 13).

According to our data, 17.4% of overall cohort exhibited a “vascular” lesion pattern. They were all part of “SSNHL + vertigo” subgroup and accounted for most patients herein included (*p* < 0.001), consistent with previous reports (29, 32). In general, these patients seemed to represent the part of the overall cohort with the worst hearing performance and prognosis, and the category with the highest WML score (*p* < 0.001), confirming the close relationship between these types of lesion pattern and vascular disease, and highlighting their poor HL at presentation (*p* = 0.001) and worse hearing recovery (*p* = 0.026). These findings are in line with animal studies on cochlear vulnerability to ischemia showing that 30 min hypoxia can induce irreversible cochlear damages (113), and also confirm previous data on the correlations among vascular disorders, severe SSNHL and worst outcome (7–10). These data should encourage further studies related to causes of ischemia and its therapy.

The prospective nature of the study with a complete vestibular assessment, along with the number of consecutive patients managed with a uniform treatment protocol, give strength to data obtained from the present study. Nevertheless, even though a substantial portion of SSNHL patients with/without vertigo seems to be related either to vascular disorders or MD, a considerable part of subjects with non-specific audio-vestibular findings still remains as unknown significance. Further clinical trials are needed to provide insights into unknown pathogenesis of the disease and establish evidence-based management. In addition, some limitations for this study exist. First, the inclusion of both “definite” and “probable” MD patients in both acute and inter-ictal stages could have affected the power of the statistical analysis. The use of well-recognized diagnostic tool for EH, such as electrocochleography and 3T brain MRI with delayed acquisition following Gadolinium infusion, could have surely helped in better differentiating MD patients from the other subgroups. Nevertheless, MD itself represents a multifactorial disease and a fluctuating disorder, resulting in heterogeneous findings. Continuous changings of inner ear function depending on the labyrinthine compartments involved and on the stages of the disease, including SC VOR-gain, cVEMPs and oVEMPs amplitudes, SN direction and other oculomotor findings, might have further ruined the data collection. Additionally, we did not include caloric test and rotatory test data to identify VOR lesions in the low frequency domain and for the midrange VOR function, respectively, even though they represent the tests mainly impaired in MD patients. Moreover, we could only test otolith pathways with AC sounds as it represents the only setting at our disposal in our institution, whereas it is well-known how bone conduction likely represents the best stimulus to obtain oVEMPs especially in elderly patients. Therefore, the applied method might have missed otolith pathology. Another shortcoming is represented by the wide range of days from the onset of symptoms and clinical evaluation (0–30). Asymmetrical recovery of the activity of different vestibular end-organs over time might have accounted for heterogenous data not fitting well established lesion patterns. Moreover, since both otolith organs receive a dual vascular supply from both the anterior vestibular artery and the CCA, it might be possible that our definition of “vascular” pattern (always including saccular involvement) might have underestimated the prevalence of other labyrinthine ischemia sparing the saccular macula. Additionally, it should be considered that a transient inner ear ischemia may cause an incomplete pattern of labyrinthine damage according to individual inner ear organ susceptibility to ischemia. Nonetheless, we did not consider the possible lesion patterns matching the venous drainage system of the inner ear and it branches, which seems to play a pivotal role in defining the areas of highest vulnerability according to recent studies (114). It should also be considered that brain-enhanced MRI was completed within 3 months after the evaluation in all patients, which represents a large time span to potentially detect CNS lesions in a timely manner; for this purpose, an early inner ear MRI might have had more suggestive value for the determination of vascular lesions. Moreover, using the Fazekas score to detect a vascular burden in these patients might have introduced potential confounders in the analysis of results, considering that WML might be hard to classify as vascular or non-vascular or caused by different etiologies (i.e., migraine, arterial hypertension) and that the time correlation of SSNHL and WML could not be established. Finally, though we prospective enrolled consecutive SSNHL patients who were managed with a uniform treatment protocol, some biases might have arisen from different salvage treatments according to personal and logistical issues.

## 5. Conclusions

SSHL is a disorder which includes various clinical scenario and results from a variety of etiologies. Our data confirm that the assessment of vestibular function represents a valuable method to explore underlying pathomechanisms as it provides additional data on the involvement of inner ear receptors, supporting further understandings in labyrinthine function. Vestibular assessment should always be pursued in case of SSNHL, irrespective to vestibular symptoms, as it could be useful in the detection of the possible underlying pathophysiological mechanisms, in orienting treatment strategies and protocols aimed at preventing further inner ear impairment, and in the prediction of hearing recovery. Nevertheless, further studies are needed to confirm the clinical relevance of vestibular assessment in SSNHL.

## Data availability statement

The original contributions presented in the study are included in the article/Supplementary material, further inquiries can be directed to the corresponding author.

## Ethics statement

The studies involving human participants were reviewed and approved by Area Vasta Emilia Nord. The patients/participants provided their written informed consent to participate in this study. Written informed consent was obtained from the individual(s) for the publication of any potentially identifiable images or data included in this article.

## Author contributions

AC and CB led the conception of the study and conducted most data acquisition, data interpretation, data analysis, and made significant contributions to the writing and editing of the manuscript. AC conducted the creation of figures. SD, MB, FL, PB, RR, and LG contribute to data acquisition. PM, SM, and EA were involved in project conception and manuscript editing. LR, AG, and GB were involved in manuscript review. All authors approved the final version of the manuscript.

## Supplementary material

Supplementary Table a Clinical-instrumental findings of the 25 patients fitting the “SSNHL no vertigo” subgroup.

Supplementary Table b Clinical-instrumental findings of the 27 patients fitting the “SSNHL + vertigo” subgroup.

Supplementary Table c Clinical-instrumental findings of the 34 patients fitting the “MD” subgroup.

Supplementary Video 1 Video-Frenzel examination of patients #42 with an instrumental lesion pattern consistent with an ischemic damage in the territory mainly supplied by the right VCA.

Supplementary Video 2 Video-Frenzel examination of patients #64 with an instrumental lesion pattern consistent with an ischemic damage in the territory mainly supplied by the right CAA.

Supplementary Video 3 Video-Frenzel examination of patients #59 with an instrumental lesion pattern consistent with an ischemic damage in the territory mainly supplied by the left IAA.
