# Supplementary material for: Vestibular assessment in sudden sensorineural hearing loss: Role in the prediction of hearing outcome and in the early detection of vascular and hydropic pathomechanisms
Source: Front Neurol. 2023 Feb 15;14:1127008. doi: 10.3389/fneur.2023.1127008 (PMC9975513; doi:10.3389/fneur.2023.1127008)
Supplement: Supplementary Table a — Clinical-instrumental findings of the 25 patients fitting the “SSNHL no vertigo” subgroup. [file Table_1.DOCX]

**Table a.** Clinical-instrumental findings of the 25 patients fitting the “SSNHL no vertigo” subgroup.

| **history** | | | | | | | **cochlear assessment** | | | | | | | **imaging** | | **vestibular assessment** | | | | | | | | | | | | |
| --- | --- | --- | --- | --- | --- | --- | --- | --- | --- | --- | --- | --- | --- | --- | --- | --- | --- | --- | --- | --- | --- | --- | --- | --- | --- | --- | --- | --- |
|  |  |  |  |  |  |  | **presenting hearing function** | | | **post-treatment hearing function** | | | | **MRI findings** | | **otolith function** | | | | **semicircular canal function** | | | **vascular category** | **video-Frenzel findings** | | | | |
| **ID** | **age** | **sex** | **side** | **n. risk factors** | **time (days)** | **vestibular symptoms** | **PTA pre (dB)** | **HL conf** | **HL degree** | **PTA post (dB)** | **PTA recovery (dB)** | **hearing recovery** | **mean % of hearing recovery** | **WML** | **Fazekas grading** | **cVEMPs AR (%)** | **cVEMPs freq tuning ipsi** | **cVEMPs freq tuning cont** | **oVEMPs AR (%)** | **HSC VOR gain** | **ASC VOR gain** | **PSC VOR gain** |  | **SN** | **PN** | **HSN** | **VIN** | **HVN** |
| **2** | 47 | M | L | 2 | 10 | no | 56,25 | LF | moderate | 41,25 | 15 | no | 41 | no | 0 | -14 | neg | neg | -21 | 0,80 | 0,76 | 0,79 | non-vasc | / | / | / | / | / |
| **10** | 73 | F | L | 1 | 17 | no | 55 | flat | moderate | 6,25 | 48,75 | complete | 100 | yes | 1 | -100 | neg | neg | 100 | 1,07 | 0,95 | 0,81 | non-vasc | / | / | down | / | / |
| **11** | 50 | F | L | 4 | 26 | no | 68,75 | flat | moderate | 68,75 | 0 | no | 0 | yes | 1 | 100 | neg | pos | 100 | 0,78 | 0,83 | 0,93 | non-vasc | / | geo | cont | / | / |
| **13** | 63 | F | L | 1 | 11 | no | 110 | flat | prof / anac | 102,5 | 7,5 | no | 8 | no | 0 | 5 | neg | neg | 100 | 0,86 | 0,82 | 0,79 | non-vasc | cont | / | / | / | / |
| **19** | 23 | F | R | 1 | 11 | no | 21,25 | LF | mild | 10 | 11,25 | complete | 100 | no | 0 | -9 | neg | neg | -100 | 1,05 | 0,74 | 0,78 | non-vasc | / | / | / | / | / |
| **21** | 34 | F | R | 2 | 4 | no | 70 | flat | severe | 11,25 | 58,75 | complete | 100 | no | 0 | -10 | neg | pos | -34 | 1,04 | 0,90 | 0,69 | non-vasc | / | / | / | / | / |
| **23** | 45 | M | L | 3 | 2 | no | 87,5 | flat | severe | 40 | 47,5 | partial | 70 | yes | 1 | -3 | neg | pos | 25 | 0,88 | 0,75 | 0,98 | non-vasc | / | apo | / | / | / |
| **24** | 69 | M | L | 3 | 5 | no | 95 | flat | prof / anac | 58,75 | 36,25 | partial | 69 | yes | 2 | 100 | neg | pos | 100 | 0,84 | 0,92 | 0,70 | non-vasc | / | / | cont | cont | / |
| **25** | 35 | M | R | 4 | 30 | no | 116,25 | flat | prof / anac | 118,75 | -2,5 | no | 0 | no | 0 | 20 | neg | neg | 13 | 1,00 | 0,85 | 0,75 | non-vasc | ipsi | / | / | / | / |
| **27** | 40 | M | R | 4 | 30 | no | 35 | flat | mild | 32,5 | 2,5 | no | 13 | no | 0 | 100 | neg | pos | 5 | 1,11 | 0,96 | 0,92 | non-vasc | / | apo | cont | / | cont |
| **29** | 60 | M | R | 3 | 22 | no | 77,5 | flat | severe | 50 | 27,5 | partial | 69 | yes | 1 | -19 | neg | neg | -100 | 0,99 | 0,97 | 0,92 | non-vasc | / | / | down | ipsi | / |
| **33** | 40 | M | R | 1 | 4 | no | 60 | LF | moderate | 66,25 | -6,25 | no | 0 | no | 0 | 3 | neg | neg | -43 | 1,07 | 0,82 | 0,71 | non-vasc | / | / | / | ipsi | / |
| **35** | 62 | F | L | 1 | 7 | no | 83,75 | flat | severe | 22,5 | 61,25 | complete | 100 | yes | 2 | 20 | neg | neg | 100 | 0,90 | 0,93 | 0,84 | non-vasc | / | / | / | / | / |
| **38** | 60 | F | L | 3 | 3 | no | 98,75 | DS | prof / anac | 70 | 28,75 | partial | 34 | yes | 1 | 33 | neg | neg | 15 | 1,14 | 0,92 | 0,76 | non-vasc | / | / | ipsi | / | / |
| **43** | 54 | M | R | 2 | 2 | no | 85 | flat | severe | 75 | 10 | partial | 20 | yes | 1 | -9 | neg | neg | -10 | 0,83 | 0,90 | 0,92 | non-vasc | / | / | / | / | / |
| **56** | 54 | M | R | 1 | 22 | no | 65 | DS | moderate | 77,5 | -12,5 | no | 0 | no | 0 | 25 | pos | neg | 6 | 0,82 | 0,89 | 0,66 | non-vasc | ipsi | / | / | / | / |
| **60** | 57 | F | R | 2 | 14 | no | 56,25 | flat | moderate | 33,75 | 22,5 | partial | 64 | yes | 1 | 5 | neg | neg | -17 | 1,12 | 1,04 | 0,81 | non-vasc | / | / | ipsi | / | / |
| **61** | 46 | M | L | 0 | 10 | no | 51,25 | DS | moderate | 30 | 21,25 | partial | 71 | no | 0 | 100 | neg | neg | 100 | 0,87 | 0,82 | 0,82 | non-vasc | / | / | / | / | / |
| **63** | 45 | M | L | 3 | 15 | no | 56,25 | DS | moderate | 50 | 6,25 | no | 19 | no | 0 | -11 | neg | neg | 100 | 0,84 | 0,89 | 0,81 | non-vasc | / | down | ipsi | ipsi | down |
| **68** | 73 | F | R | 2 | 30 | no | 70 | DS | severe | 50 | 20 | partial | 70 | yes | 1 | 0 | neg | neg | -6 | 1,19 | 0,87 | 0,86 | non-vasc | / | / | / | / | / |
| **71** | 65 | M | L | 2 | 30 | no | 55 | DS | moderate | 46,25 | 8,75 | no | 88 | yes | 2 | 5 | neg | neg | 16 | 1,02 | 0,75 | 0,87 | non-vasc | / | apo | / | ipsi | ipsi |
| **73** | 74 | F | L | 2 | 3 | no | 72,5 | flat | severe | 72,5 | 0 | no | 0 | yes | 1 | -17 | neg | neg | 33 | 0,83 | 0,78 | 0,80 | non-vasc | / | geo | down | / | / |
| **76** | 69 | M | L | 1 | 30 | no | 56,25 | flat | moderate | 60 | -3,75 | no | 0 | yes | 1 | 100 | neg | neg | 20 | 0,92 | 0,96 | 0,82 | non-vasc | / | / | down | down | / |
| **83** | 58 | F | L | 0 | 4 | no | 55 | DS | moderate | 36,25 | 18,75 | complete | 68 | yes | 1 | 100 | neg | neg | -20 | 0,80 | 0,84 | 0,89 | non-vasc | / | / | / | / | ipsi |
| **86** | 61 | M | R | 2 | 16 | no | 97,5 | flat | prof / anac | 80 | 17,5 | no | 21 | yes | 1 | 100 | neg | neg | -60 | 1,11 | 0,93 | 0,90 | non-vasc | cont | / | / | / | / |

**Abbreviations:** Apo: apogeotropic. AR: asymmetry ratio. ASC: anterior semicircular canal. Conf: configuration. Cont: contralesional. cVEMPs: cervical vestibular evoked myogenic potentials. Down: downbeating. DS: down-sloping. F: female. Freq: frequency. Geo: geotropic. HL: hearing loss. HSC: horizontal semicircular canal. HSN: head-shaking nystagmus. HVN: hyperventilation nystagmus. Ipsi: ipsilesional. L: left. LF: low-frequency. M: male. MRI: magnetic resonance imaging. Neg: negative. Non-vasc: non-vascular. Ny: nystagmus. oVEMPs: ocular vestibular evoked myogenic potentials. PN: positional nystagmus. Pos: positive. Post: post-treatment. Pre: presenting. Prof / anac: profound / anacusis. PSC: posterior semicircular canal. PTA: pure tone average. R: right. SN: spontaneous nystagmus. SSNHL: sudden sensorineural hearing loss. VIN: vibration-induced nystagmus. VOR: vestibulo-ocular reflex. WML: white matter lesions.

**Table b.** Clinical-instrumental findings of the 27 patients fitting the “SSNHL + vertigo” subgroup.

| **history** | | | | | | | **cochlear assessment** | | | | | | | **imaging** | | **vestibular assessment** | | | | | | | | | | | | |
| --- | --- | --- | --- | --- | --- | --- | --- | --- | --- | --- | --- | --- | --- | --- | --- | --- | --- | --- | --- | --- | --- | --- | --- | --- | --- | --- | --- | --- |
|  |  |  |  |  |  |  | **presenting hearing function** | | | **post-treatment hearing function** | | | | **MRI findings** | | **otolith function** | | | | **semicircular canal function** | | | **vascular category** | **video-Frenzel findings** | | | | |
| **ID** | **age** | **sex** | **side** | **n. risk factors** | **time (days)** | **vestibular symptoms** | **PTA pre (dB)** | **HL conf** | **HL degree** | **PTA post (dB)** | **PTA recovery (dB)** | **hearing recovery** | **mean % of hearing recovery** | **WML** | **Fazekas grading** | **cVEMPs AR (%)** | **cVEMPs freq tuning ipsi** | **cVEMPs freq tuning cont** | **oVEMPs AR (%)** | **HSC VOR gain** | **ASC VOR gain** | **PSC VOR gain** |  | **SN** | **PN** | **HSN** | **VIN** | **HVN** |
| **4** | 45 | M | L | 3 | 10 | yes | 110 | DS | prof / anac | 77,5 | 32,5 | partial | 35 | no | 0 | 35 | pos | pos | 100 | 0,36 | 0,69 | 0,42 | vasc | cont | / | / | / | ipsi |
| **6** | 49 | M | R | 0 | 12 | yes | 53,75 | flat | moderate | 27,5 | 26,25 | complete | 70 | no | 0 | -18 | pos | pos | -40 | 1,00 | 0,95 | 0,88 | non-vasc | / | / | / | / | / |
| **7** | 67 | F | L | 3 | 5 | yes | 82,5 | flat | severe | 48,75 | 33,75 | partial | 48 | no | 3 | 38 | neg | neg | 100 | 0,73 | 0,49 | 0,69 | vasc | / | apo | cont | cont | cont |
| **8** | 70 | F | R | 4 | 6 | yes | 81,25 | DS | severe | 60 | 21,25 | no | 32 | yes | 0 | 34 | neg | neg | 100 | 1,13 | 0,87 | 0,57 | vasc | cont | / | / | / | / |
| **14** | 75 | M | R | 5 | 11 | yes | 100 | flat | prof / anac | 75 | 25 | partial | 29 | yes | 2 | 100 | neg | neg | 100 | 0,98 | 0,88 | 0,78 | non-vasc | / | / | / | / | / |
| **17** | 64 | M | R | 2 | 24 | yes | 87,5 | flat | severe | 10 | 77,5 | complete | 100 | yes | 1 | 100 | neg | neg | 5 | 0,96 | 0,73 | 0,99 | non-vasc | / | / | / | ipsi | / |
| **20** | 55 | F | L | 1 | 21 | yes | 28,75 | flat | mild | 11,25 | 17,5 | complete | 100 | no | 0 | 19 | neg | neg | 27 | 0,88 | 0,92 | 0,96 | non-vasc | / | / | down | / | / |
| **26** | 51 | M | L | 3 | 8 | yes | 107,5 | flat | prof / anac | 118,75 | -11,25 | no | 0 | no | 2 | 100 | neg | neg | 15 | 0,71 | 0,72 | 0,80 | non-vasc | cont | bigeo | / | ipsi | / |
| **31** | 45 | M | L | 2 | 10 | yes | 95 | flat | prof / anac | 72,5 | 22,5 | no | 26 | yes | 1 | 67 | neg | pos | 42 | 0,80 | 0,92 | 0,34 | vasc | cont | / | ipsi | / | / |
| **34** | 71 | M | L | 4 | 10 | yes | 112,5 | flat | prof / anac | 90 | 22,5 | partial | 26 | yes | 3 | 35 | neg | neg | 9 | 0,94 | 0,90 | 0,61 | vasc | cont | / | / | / | / |
| **39** | 38 | M | L | 1 | 15 | yes | 48,75 | DS | moderate | 35 | 13,75 | complete | 65 | no | 0 | 17 | neg | neg | -100 | 0,78 | 0,73 | 0,84 | non-vasc | / | biapo | / | cont | cont |
| **40** | 64 | F | R | 3 | 3 | yes | 105 | flat | prof / anac | 120 | -15 | no | 0 | yes | 1 | 100 | neg | pos | 39 | 0,49 | 0,34 | 0,24 | vasc | cont | / | / | / | / |
| **41** | 68 | M | R | 5 | 30 | yes | 92,5 | DS | prof / anac | 90 | 2,5 | no | 6 | yes | 3 | 100 | neg | pos | -100 | 0,80 | 0,80 | 0,40 | vasc | / | biapo | cont | ipsi | / |
| **42** | 54 | F | R | 4 | 4 | yes | 48,75 | DS | moderate | 45 | 3,75 | no | 13 | no | 0 | 100 | neg | neg | 29 | 0,92 | 0,98 | 0,42 | vasc | cont | / | / | ipsi | / |
| **44** | 65 | F | R | 2 | 4 | yes | 97,5 | DS | prof / anac | 67,5 | 30 | partial | 48 | no | 0 | 100 | neg | neg | -29 | 0,85 | 0,70 | 0,65 | vasc | cont | BPPV | / | / | / |
| **46** | 84 | F | L | 4 | 9 | yes | 61,25 | DS | moderate | 55 | 6,25 | no | 16 | yes | 2 | 100 | neg | neg | 100 | 0,95 | 0,74 | 0,77 | non-vasc | ipsi | / | down | / | / |
| **47** | 74 | M | L | 4 | 6 | yes | 112,5 | flat | prof / anac | 120 | -7,5 | no | 0 | yes | 2 | 35 | pos | neg | -4 | 0,97 | 0,94 | 0,87 | non-vasc | cont | / | / | ipsi | / |
| **51** | 22 | M | L | 1 | 30 | yes | 45 | DS | mod | 37,5 | 7,5 | partial | 40 | no | 0 | 6 | pos | neg | 6 | 0,83 | 0,82 | 0,89 | non-vasc | / | / | / | / | / |
| **54** | 74 | M | R | 3 | 25 | yes | 71,25 | DS | severe | 37,5 | 33,75 | partial | 73 | yes | 2 | 100 | neg | neg | 41 | 0,40 | 0,36 | 0,15 | vasc | cont | / | / | / | ipsi |
| **59** | 76 | M | L | 3 | 13 | yes | 76,25 | flat | severe | 63,75 | 12,5 | partial | 29 | yes | 2 | 100 | neg | neg | 100 | 0,27 | 0,19 | 0,68 | vasc | cont | / | / | / | / |
| **64** | 60 | M | R | 2 | 20 | yes | 83,75 | DS | severe | 83,75 | 0 | no | 0 | yes | 2 | 100 | neg | neg | 33 | 0,97 | 0,91 | 0,60 | vasc | down | / | / | cont | / |
| **69** | 71 | F | L | 3 | 0 | yes | 73,75 | flat | severe | 88,75 | -15 | no | 0 | no | 0 | 100 | neg | neg | 100 | 0,89 | 0,89 | 0,67 | vasc | / | apo | / | cont | / |
| **72** | 50 | M | R | 0 | 30 | yes | 92,5 | flat | prof / anac | 71,25 | 21,25 | partial | 27 | no | 0 | 12 | neg | neg | 100 | 0,97 | 0,81 | 0,82 | non-vasc | cont | / | down | / | / |
| **77** | 50 | F | R | 1 | 4 | yes | 120 | flat | prof / anac | 120 | 0 | no | 0 | no | 0 | 10 | neg | neg | 7 | 1,07 | 0,87 | 0,80 | non-vasc | / | BPPV | cont | / | / |
| **78** | 75 | F | L | 1 | 30 | yes | 102,5 | DS | prof / anac | 115 | -12,5 | no | 0 | yes | 3 | 100 | neg | neg | 100 | 0,73 | 0,60 | 0,53 | vasc | / | BPPV | cont | cont | / |
| **81** | 74 | M | R | 0 | 30 | yes | 60 | flat | moderate | 60 | 0 | no | 0 | yes | 1 | 100 | neg | neg | 100 | 1,03 | 0,80 | 0,66 | vasc | cont | / | / | / | / |
| **84** | 46 | M | L | 2 | 3 | yes | 52,5 | DS | moderate | 23,75 | 28,75 | complete | 100 | no | 0 | 0 | neg | neg | -23 | 0,82 | 0,98 | 0,90 | non-vasc | ipsi | / | / | / | / |

**Abbreviations:** Apo: apogeotropic. AR: asymmetry ratio. Biapo: biapogeotropic. Bigeo: bigeotropic. BPPV: benign paroxysmal positional vertigo. ASC: anterior semicircular canal. Conf: configuration. Cont: contralesional. cVEMPs: cervical vestibular-evoked myogenic potentials. Down: downbeating. DS: down-sloping. F: female. Freq: frequency. HL: hearing loss. HSC: horizontal semicircular canal. HSN: head-shaking nystagmus. HVN: hyperventilation nystagmus. Ipsi: ipsilesional. L: left. LF: low-frequency. M: male. MRI: magnetic resonance imaging. Neg: negative. Non-vasc: non-vascular. Ny: nystagmus. oVEMPs: ocular vestibular-evoked myogenic potentials. PN: positional nystagmus. Pos: positive. Post: post-treatment. Pre: presenting. Prof / anac: profound / anacusis. PSC: posterior semicircular canal. PTA: pure tone average. R: right. SN: spontaneous nystagmus. SSNHL: sudden sensorineural hearing loss. Vasc: vascular. VIN: vibration-induced nystagmus. VOR: vestibulo-ocular reflex. WML: white matter lesions.

**Table c.** Clinical-instrumental findings of the 34 patients fitting the “MD” subgroup.

| **history** | | | | | | | **cochlear assessment** | | | | | | | **imaging** | | **vestibular assessment** | | | | | | | | | | | | |
| --- | --- | --- | --- | --- | --- | --- | --- | --- | --- | --- | --- | --- | --- | --- | --- | --- | --- | --- | --- | --- | --- | --- | --- | --- | --- | --- | --- | --- |
|  |  |  |  |  |  |  | **presenting hearing function** | | | **post-treatment hearing function** | | | | **MRI findings** | | **otolith function** | | | | **semicircular canal function** | | | **vascular category** | **video-Frenzel findings** | | | | |
| **ID** | **age** | **sex** | **side** | **n. risk factors** | **time (days)** | **vestibular symptoms** | **PTA pre (dB)** | **HL conf** | **HL degree** | **PTA post (dB)** | **PTA recovery (dB)** | **hearing recovery** | **mean % of hearing recovery** | **WML** | **Fazekas grading** | **cVEMPs AR (%)** | **cVEMPs freq tuning ipsi** | **cVEMPs freq tuning cont** | **oVEMPs AR (%)** | **HSC VOR gain** | **ASC VOR gain** | **PSC VOR gain** |  | **SN** | **PN** | **HSN** | **VIN** | **HVN** |
| **1** | 47 | F | R | 4 | 4 | no | 38,75 | LF | mild | 10 | 28,75 | complete | 100 | no | 0 | -36 | neg | neg | 56 | 1,01 | 0,83 | 0,82 | non-vasc | / | apo | cont | / | cont |
| **3** | 27 | F | L | 1 | 30 | no | 42,5 | LF | moderate | 20 | 22,5 | complete | 69 | yes | 1 | 6 | pos | neg | 100 | 0,89 | 0,84 | 0,70 | non-vasc | ipsi | / | / | / | / |
| **5** | 32 | F | L | 0 | 30 | yes | 50 | LF | moderate | 40 | 10 | no | 25 | yes | 1 | -15 | neg | neg | 3 | 0,80 | 0,80 | 0,82 | non-vasc | / | / | cont | / | / |
| **9** | 52 | F | R | 0 | 9 | yes | 53,75 | LF | moderate | 23,75 | 30 | partial | 73 | yes | 2 | 37 | neg | neg | 5 | 0,96 | 0,88 | 0,94 | non-vasc | / | / | / | / | / |
| **12** | 36 | M | L | 2 | 3 | no | 55 | LF | moderate | 11,25 | 43,75 | complete | 100 | yes | 1 | -4 | neg | neg | 1 | 0,86 | 0,87 | 0,80 | non-vasc | / | / | / | / | / |
| **15** | 47 | M | L | 3 | 2 | no | 65 | flat | moderate | 66,25 | -1,25 | no | 0 | no | 0 | 22 | neg | neg | 36 | 0,96 | 0,85 | 0,86 | non-vasc | ipsi | / | / | / | / |
| **16** | 65 | M | L | 3 | 24 | no | 52,5 | flat | moderate | 35 | 17,5 | partial | 64 | yes | 1 | 58 | neg | neg | 45 | 1,09 | 1,02 | 0,89 | non-vasc | / | biapo | ipsi | / | / |
| **18** | 67 | F | L | 2 | 17 | yes | 56,25 | DS | moderate | 55 | 1,25 | no | 3 | no | 0 | 100 | neg | neg | 19 | 1,06 | 0,68 | 0,90 | non-vasc | / | / | / | cont | / |
| **22** | 76 | F | L | 3 | 5 | yes | 77,5 | flat | severe | 57,5 | 20 | partial | 36 | yes | 2 | 3 | neg | neg | -13 | 0,78 | 0,38 | 0,82 | non-vasc | ipsi | / | / | cont | / |
| **28** | 31 | F | L | 0 | 0 | yes | 35 | LF | mild | 11,25 | 23,75 | complete | 100 | no | 0 | -24 | neg | neg | 100 | 0,88 | 0,87 | 0,78 | non-vasc | cont | / | / | / | / |
| **30** | 76 | M | L | 2 | 21 | yes | 55 | DS | moderate | 71,25 | -16,25 | no | 54 | no | 0 | 100 | pos | neg | 100 | 0,68 | 0,83 | 0,79 | non-vasc | / | / | / | cont | / |
| **32** | 52 | F | L | 1 | 1 | no | 52,5 | LF | moderate | 12,5 | 40 | complete | 100 | yes | 1 | 17 | pos | pos | 40 | 0,90 | 0,83 | 0,90 | non-vasc | / | up | ipsi | / | / |
| **36** | 39 | F | R | 2 | 11 | no | 47,5 | flat | moderate | 35 | 12,5 | partial | 67 | no | 0 | -3 | neg | neg | 100 | 0,99 | 0,86 | 0,75 | non-vasc | / | / | ipsi | / | / |
| **37** | 60 | M | L | 5 | 26 | yes | 57,5 | flat | moderate | 58,75 | -1,25 | no | 0 | yes | 1 | 100 | neg | neg | 100 | 0,73 | 0,59 | 0,80 | non-vasc | ipsi | / | / | / | / |
| **45** | 78 | M | L | 5 | 19 | yes | 65 | flat | moderate | 55 | 10 | partial | 30 | no | 2 | 100 | pos | pos | 100 | 0,99 | 0,84 | 0,71 | non-vasc | ipsi | / | / | / | / |
| **48** | 45 | F | L | 3 | 10 | yes | 38,75 | LF | mild | 26,25 | 12,5 | partial | 59 | no | 0 | 19 | pos | pos | 10 | 0,87 | 0,71 | 0,95 | non-vasc | ipsi | / | / | / | / |
| **49** | 60 | M | L | 1 | 9 | yes | 53,75 | LF | moderate | 31,25 | 22,5 | partial | 69 | yes | 1 | 4 | neg | neg | -34 | 0,97 | 0,76 | 0,72 | non-vasc | cont | / | / | / | / |
| **50** | 57 | M | R | 2 | 7 | yes | 41,25 | LF | moderate | 11,25 | 30 | complete | 100 | yes | 1 | 0 | pos | neg | 5 | 0,97 | 0,74 | 0,90 | non-vasc | down | / | / | / | / |
| **52** | 69 | M | L | 2 | 30 | no | 52,5 | flat | moderate | 52,5 | 0 | no | 0 | yes | 0 | -15 | pos | neg | 8 | 0,97 | 0,72 | 0,88 | non-vasc | / | / | / | / | / |
| **53** | 33 | F | R | 1 | 8 | no | 55 | flat | moderate | 11,25 | 43,75 | complete | 100 | no | 0 | 18 | pos | pos | 2 | 0,96 | 0,72 | 0,81 | non-vasc | / | / | cont | cont | / |
| **55** | 55 | M | R | 2 | 15 | no | 48,75 | LF | moderate | 36,25 | 12,5 | partial | 42 | no | 0 | 5 | neg | neg | 100 | 0,99 | 0,88 | 0,83 | non-vasc | / | / | / | / | / |
| **57** | 70 | M | L | 3 | 20 | yes | 55 | LF | moderate | 41,25 | 13,75 | partial | 37 | yes | 1 | 100 | neg | pos | 100 | 0,93 | 0,83 | 0,85 | non-vasc | / | / | / | / | / |
| **58** | 58 | M | R | 3 | 13 | no | 32,5 | flat | mild | 18,75 | 13,75 | partial | 80 | no | 0 | 51 | pos | neg | 33 | 0,82 | 0,84 | 0,83 | non-vasc | up | / | / | / | / |
| **62** | 36 | F | L | 1 | 30 | no | 25 | flat | mild | 10 | 15 | complete | 100 | no | 0 | -100 | neg | pos | 4 | 0,91 | 0,83 | 0,81 | non-vasc | / | / | down | / | / |
| **65** | 42 | F | L | 0 | 30 | yes | 28,75 | LF | mild | 32,5 | -3,75 | no | 0 | no | 0 | -17 | neg | neg | -20 | 0,86 | 0,78 | 0,84 | non-vasc | up | / | cont | / | / |
| **66** | 51 | M | L | 3 | 8 | yes | 66,25 | LF | moderate | 20 | 46,25 | complete | 84 | yes | 2 | 100 | neg | neg | 100 | 0,75 | 0,80 | 0,88 | non-vasc | ipsi | / | cont | cont | / |
| **67** | 57 | M | L | 2 | 4 | no | 116,25 | DS | prof / anac | 106,25 | 10 | no | 14 | no | 0 | -100 | neg | neg | -11 | 0,92 | 0,60 | 0,96 | non-vasc | ipsi | / | down | / | / |
| **70** | 61 | M | L | 3 | 10 | yes | 57,5 | flat | moderate | 61,25 | -3,75 | no | 0 | yes | 1 | 100 | neg | neg | -100 | 0,76 | 0,80 | 1,00 | non-vasc | / | / | cont | cont | / |
| **74** | 47 | F | L | 1 | 30 | no | 32,5 | LF | mild | 13,75 | 18,75 | complete | 88 | no | 0 | 2 | neg | neg | -33 | 1,10 | 0,76 | 0,82 | non-vasc | / | / | cont | / | cont |
| **75** | 66 | M | R | 3 | 11 | no | 70 | flat | severe | 35 | 35 | complete | 82 | yes | 1 | 100 | neg | neg | 100 | 1,06 | 0,98 | 0,98 | non-vasc | / | / | cont | / | / |
| **79** | 48 | F | R | 1 | 2 | yes | 61,25 | LF | moderate | 22,5 | 38,75 | partial | 84 | no | 0 | 10 | neg | neg | -14 | 1,20 | 0,96 | 0,88 | non-vasc | / | / | ipsi | / | ipsi |
| **80** | 29 | F | R | 1 | 5 | no | 43,75 | LF | moderate | 13,75 | 30 | complete | 100 | no | 0 | 6 | pos | neg | -10 | 0,93 | 0,84 | 0,73 | non-vasc | / | / | / | / | / |
| **82** | 60 | F | R | 2 | 15 | yes | 36,25 | LF | mild | 10 | 26,25 | complete | 100 | yes | 1 | -16 | neg | neg | 100 | 1,29 | 0,79 | 0,80 | non-vasc | cont | / | / | ipsi | / |
| **85** | 69 | M | R | 2 | 12 | yes | 43,75 | flat | moderate | 21,25 | 22,5 | complete | 100 | no | 0 | 30 | neg | neg | 8 | 1,11 | 0,83 | 0,69 | non-vasc | / | apo | down | / | / |

**Abbreviations:** Apo: apogeotropic. AR: asymmetry ratio. Biapo: biapogeotropic. ASC: anterior semicircular canal. Conf: configuration. Cont: contralesional. cVEMPs: cervical vestibular-evoked myogenic potentials. Down: downbeating. DS: down-sloping. F: female. Freq: frequency. HL: hearing loss. HSC: horizontal semicircular canal. HSN: head-shaking nystagmus. HVN: hyperventilation nystagmus. Ipsi: ipsilesional. L: left. LF: low-frequency. M: male. MD: Menière’s disease. MRI: magnetic resonance imaging. Neg: negative. Non-vasc: non-vascular. Ny: nystagmus. oVEMPs: ocular vestibular-evoked myogenic potentials. PN: positional nystagmus. Pos: positive. Post: post-treatment. Pre: presenting. Prof / anac: profound / anacusis. PSC: posterior semicircular canal. PTA: pure tone average. R: right. SN: spontaneous nystagmus. Up: upbeating. VIN: vibration-induced nystagmus. VOR: vestibulo-ocular reflex. WML: white matter lesions.
